# Analysis of intraspecies diversity reveals a subset of highly variable plant immune receptors and predicts their binding sites

**DOI:** 10.1093/plcell/koab013

**Published:** 2021-01-25

**Authors:** Daniil M Prigozhin, Ksenia V Krasileva

**Affiliations:** Berkeley Center for Structural Biology, Molecular Biophysics and Integrated Bioimaging Division, Lawrence Berkeley National Laboratory, Berkeley, CA 94720, USA; Department of Plant and Microbial Biology, University of California, Berkeley, CA 94720, USA

## Abstract

The evolution of recognition specificities by the immune system depends on the generation of receptor diversity and on connecting the binding of new antigens with the initiation of downstream signaling. In plant immunity, the innate Nucleotide-Binding Leucine-Rich Repeat (NLR) receptor family enables antigen binding and immune signaling. In this study, we surveyed the NLR complements of 62 ecotypes of *Arabidopsis thaliana* and 54 lines of *Brachypodium distachyon* and identified a limited number of NLR subfamilies that show high allelic diversity. We show that the predicted specificity-determining residues cluster on the surfaces of Leucine-Rich Repeat domains, but the locations of the clusters vary among NLR subfamilies. By comparing NLR phylogeny, allelic diversity, and known functions of the Arabidopsis NLRs, we formulate a hypothesis for the emergence of direct and indirect pathogen-sensing receptors and of the autoimmune NLRs. These findings reveal the recurring patterns of evolution of innate immunity and can inform NLR engineering efforts.

## Teaser

NLR immune receptor complements of 62 ecotypes of *A. thaliana* and 54 lines of *B. distachyon* help identify highly variable NLR subfamilies responsible for the generation of new receptor specificities.

##  

**Figure:**
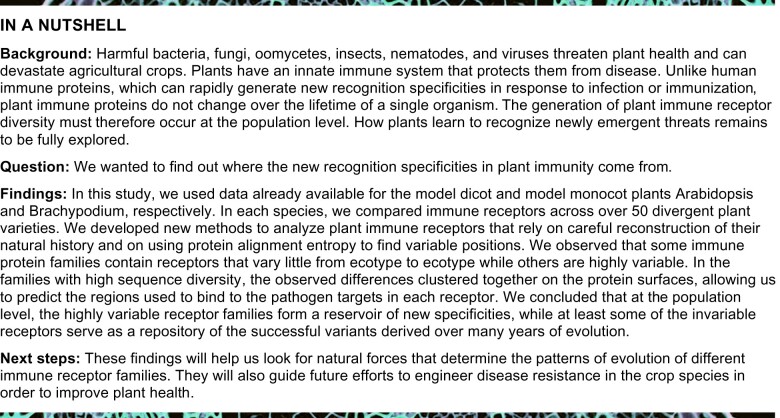


## Introduction

Plants lack the adaptive immunity of vertebrates. With their immune receptor specificities encoded in the germline, plants can achieve remarkable receptor diversity at the population level (Bakker et al., 2006). The mechanisms that generate this diversity and select for useful (and against deleterious) receptor variants are thus of great importance to both basic science and crop improvement (Dangl et al., 2013). Ongoing efforts at pan-genome sequencing of both model and crop species reveal the intraspecies diversity of plant immune receptors, their natural history, mechanisms of action, and the evolutionary forces that shape plant immunity (Gordon et al., 2017; Stam et al., 2019a, 2019b; Van de Weyer et al., 2019; Seong et al., 2020).

Two types of plant immune receptors form the basis of pathogen recognition: extracellular receptors, including receptor-like kinases (RLK) and receptor-like proteins (RLP); and intracellular Nucleotide-binding Leucine-Rich Repeat (NLR) proteins (Dangl et al., 2013). While RLKs and RLPs monitor the extracellular environments of plants, NLRs are cell death-executing receptors that are shared across the plant and animal kingdoms (Jones et al., 2016). Plant NLRs are typically composed of three domains, including a central nucleotide-binding (NB-ARC) domain that mediates receptor oligomerization upon activation, the C-terminal Leucine-Rich Repeat (LRR) domain that defines receptor specificity, and one of three N-terminal domains: Resistance To Powdery Mildew 8 (RPW8), Coiled-Coil (CC), or Toll/Interleukin-1 Receptor homology (TIR) domains, which mediate the immune effector function. NLRs are divided into three monophyletic classes based on the N-terminal domains and their evolutionary origin: RPP8-NLR (RNL), CC-NLR (CNL), and TIR-NLR (TNL) (Shao et al., 2016).

NLRs can function as sensors or signal transducers (helpers; Wu et al., 2017). For example, all RNL genes are thought to be helpers (Jubic et al., 2019), while TNLs and CNLs can fulfill either function. Sensor NLRs recognize pathogens using three main modes: (1) direct binding to the pathogen-derived effector molecules; (2) indirect recognition of effector activities on other plant proteins, and (3) recognition of modifications to a noncanonical integrated domain of the NLR, which acts as a bait for the effector (Cesari, 2018). The recognition mode of a given sensor NLR is likely to have a large effect on the evolutionary pressure it experiences. Indirect recognition NLRs likely undergo balancing or purifying selection based on the monitoring of conserved effector activity. In contrast, effector recognition upon direct binding likely requires NLRs to adapt rapidly to keep track of easy-to-mutate effector surface residues. Among the best studied NLRs that directly bind pathogen-derived effectors are the flax (*Linum usitatissimum*) L genes (Ellis et al., 2007; Catanzariti et al., 2010), the MLA/Sr50 locus in barley (*Hordeum vulgare*) and wheat (*Triticum* spp.; Chen et al., 2017; Saur et al., 2019), and the *Recognition of Peronospora Parasitica 1* (*RPP1*) genes in Arabidopsis (Krasileva et al., 2010; Goritschnig et al., 2016). Their effector targets are structurally diverse, suggesting that the current recognition specificities of individual alleles are recently derived, rather than ancestral.

The continuous generation of diversity in sensor NLRs is required to provide protection from diverse pathogens and is thought to result from divergent (diversifying) selection and a birth-and-death process acting on NLR gene clusters (Michelmore and Meyers, 1998). NLRs diversify through copy number variation, recombination, gene conversion, gene fusion, and point mutations (Baggs et al., 2017). In a subset of NLRs, these mechanisms combine to produce an astounding array of alleles (Bakker et al., 2006; Ding et al., 2007). Not unexpectedly, such diversity comes at a price. Hybrid necrosis has been observed widely in inbreeding and outcrossing plants in both cultivated and wild populations and can be considered as a plant version of autoimmunity (Bomblies, 2009). Hybrid necrosis occurs due to a mismatch between immune receptor variants and other plant genes, leading to autoimmune recognition, as exemplified by Dangerous Mix genes in *Arabidopsis thaliana* (Bomblies et al., 2007; Chae et al., 2014; Atanasov et al., 2018) and *Ne* genes in wheat (Zhang et al., 2016). Tomato (*Solanum lycopersicum*) *Cf-2* is an example of a non-NLR immune receptor that shows this phenotype (Kruger, 2002; Santangelo et al., 2003). These negative interactions revealed in crosses are likely only a small fraction of the cost of derivation of new immune specificities in the presence of the whole intracellular plant proteome.

Cross-species phylogenetic analyses of the NLR gene family have provided important insights into NLR evolution. A combined phylogeny of maize (*Zea mays*), sorghum (*Sorghum bicolor*), brachypodium, and rice (*Oryza sativa*) NLRs was used to identify recently derived NLR immune specificities against rice blast disease (Yang et al., 2013). An expansion of a network of helper and sensor NLRs was identified across asterids in which a set of diverse sensors signal through a redundant set of helpers that show reduced diversity (Wu et al., 2017). Phylogenetic analyses in grasses identified major integration clades of NLRs that incorporate additional domains that serve as baits for pathogens (Bailey et al., 2018). In view of the recent progress in elucidating the intra-species NLR complements of both model and nonmodel plants (Gordon et al., 2017; Stam et al., 2019a, 2019b; Van de Weyer et al., 2019; Seong et al., 2020), a systematic analysis is needed to uncover the relationships between NLR phylogeny, mode of recognition, and the amount of allelic diversity.

The recent elucidation of both the pre-activation monomeric and activated resistosome-forming conformations of ZAR1, an indirect recognition CNL, dramatically improved our understanding of both target binding and the receptor activation mechanisms of NLRs (Wang et al., 2019b, 2019a). The structures of Roq1 and RPP1, both direct binders, in complex with their targets, were recently revealed (Ma et al., 2020; Martin et al., 2020), further shedding light on LRR and post-LRR domain-dependent target recognition and downstream TIR domain activation. While more NLR structures are likely to be revealed in the future, structure determination efforts will likely lag behind the pan-genome sequencing due to the cost and difficulty of the experiments involved. Therefore, the prediction of the mode of recognition and specificity-determining residues of NLRs based on sequence data is an attractive direction that is yet to be fully explored. The idea that highly variable residues determine immune receptor specificity predates the elucidation of the first antibody structure by 3 years (Kabat, 1970). In the subsequent decades, several measures of amino acid diversity were advanced. Shannon entropy, which originated in information theory, is given by the formula:

$$H=\;-\sum_{i=1}^{20}p_{i}\log_{2}p_{i}$$

where *p_i_* is the fraction of 1 of the 20 amino acids in a column of a protein sequence alignment. This measure was first applied to study residues that determine antibody and T-cell receptor specificity (Shenkin et al., 1991; Stewart et al., 1997). High entropy values correlate strongly with surface exposure and hydrophilic character (Liao et al., 2005) and can be used to predict rapidly evolving ligand-binding sites (Magliery and Regan, 2005). In addition to B- and T-cell receptors, entropy-based measures have been applied to identify binding sites in TRP repeat proteins, ankyrin repeat proteins, Zn-finger transcription factors, and G protein-coupled receptors (Magliery and Regan, 2005; Sanders et al., 2011).

In the current study, we used phylogenetic analyses to group Arabidopsis and Brachypodium NLRs into near allelic series and applied Shannon entropy analyses of protein alignments to define highly variable NLRs (hvNLRs) and their candidate specificity-determining residues. Our results show that, depending on the ecotype, 15–35 Arabidopsis NLRs belong to rapidly diversifying families. These families are distributed in the NLR phylogeny among both CC- and TIR-containing NLRs and encompass the known Dangerous Mix NLRs. We further show that in the hvNLRs, the highly variable residues identified by Shannon entropy cluster on the surface of the LRR domain and contain surface-exposed hydrophobic residues, thus identifying likely binding sites. The exact location of the putative binding sites on the LRR surface is not conserved across different NLRs. Based on the phylogenetic distribution of hvNLRs, we formulate a hypothesis regarding the origin of indirect recognition sensor NLRs. When applied to *Brachypodium distachyon* pan-genome, our methods reveal a similarly dispersed phylogenetic distribution of hvNLRs in this model grass species. Collectively, our results reveal the origins of novel recognition specificities in NLR innate immune receptors and the common patterns in the evolution of innate immunity.

## Results

### Arabidopsis NLRome shows variable rates of NLR diversification

**Figure 1:**
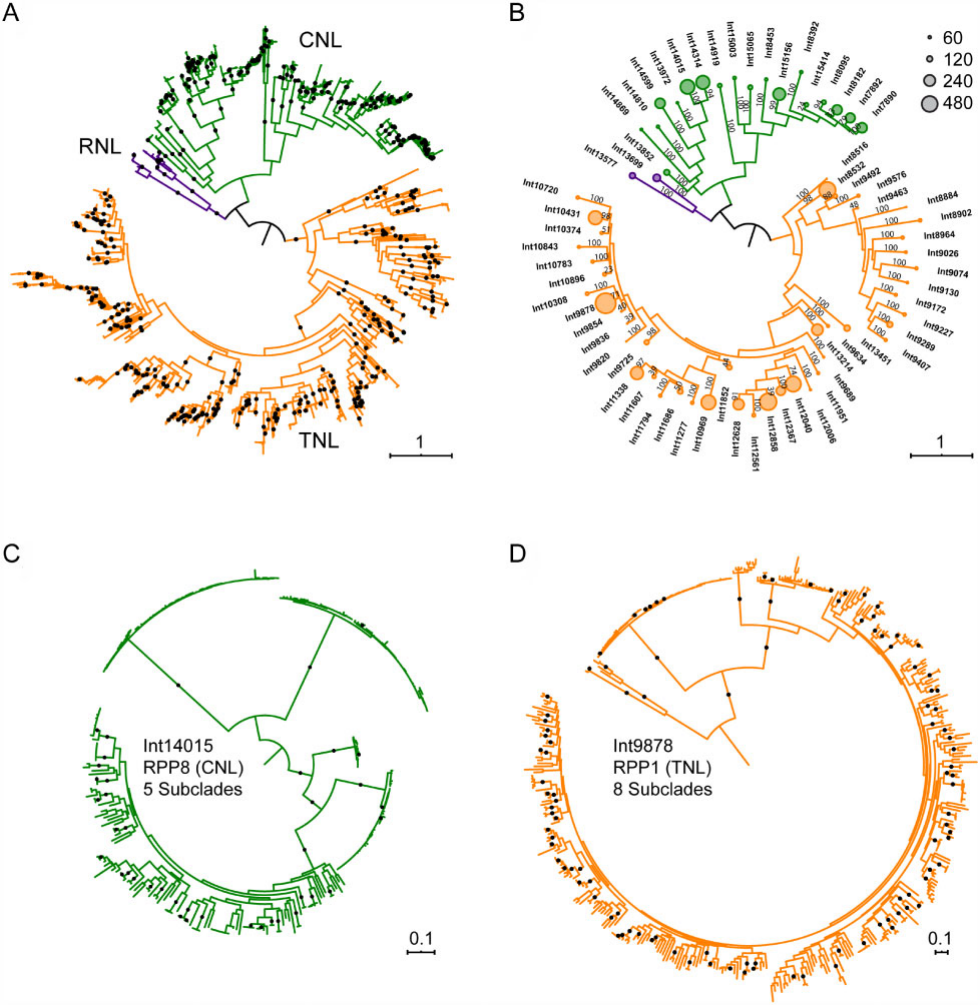
Phylogenetic analyses of Arabidopsis pan-NLRome. A, Maximum likelihood tree for 7,818 Arabidopsis NB-ARC sequences rooted on a branch connecting TNL and non-TNL clades. Ninety-nine percent or better bootstrap values are shown as dots. B, Same tree as in (A) partitioned into 65 initial clades, with circle radius proportional to clade size, and indicating bootstrap support for each clade. C, Int14015 clade tree (rooted midpoint) based on a full-length alignment of the clade sequences. Ninety-nine percent or better bootstrap values are shown as dots. D, Int9878 clade ML tree (rooted midpoint) based on a full-length alignment of the clade sequences. Ninety-nine percent or better bootstrap values are shown as dots; branch length represents the number of substitutions per site.

The recent elucidation of the NLR complements of over 60 accessions of the model plant *A. thaliana* (Van de Weyer et al., 2019) provided a unique opportunity to examine rapidly evolving clades of Arabidopsis NLRs. The unique advantage of the Arabidopsis dataset is the ability to correlate observed diversity to known functional classes of the extensively characterized NLRs. Previous NLRome analyses of this dataset were performed using OrthoMCL followed by orthogroup refinement. While these analyses provided a valuable basis for global analyses of selection pressures, they did not produce robust allelic series for each gene. This is likely due to the divergent rates of diversification across NLRs, which complicate orthogroup assignment. To circumvent this challenge, we adopted a phylogeny-based approach. To group NLRs into near allelic series, we first built a unified phylogeny of all NLRs based on their shared nucleotide-binding domain (Figure 1A). This tree contained 7,818 NB-ARC sequences that had >70% coverage across the NB-ARC domain and represented 7,716 NLR genes, including 168 NB-ARC sequences of NLRs from the reference Arabidopsis Col-0 assembly. Even though the N-terminal domains were not included in the analysis, this phylogeny clearly split into clades corresponding to the three canonical architectures: RPW8, CC, and TIR domain-containing NLRs (Shao et al., 2016; Tamborski and Krasileva, 2020). We arbitrarily placed the root of the tree between TNL and non-TNL NLRs to simplify downstream analyses.

We split the overall phylogeny into 65 clades based on clade size (40–500 sequences) and bootstrap support. Of these, 43 clades had bootstrap scores of 100, 12 additional clades had bootstrap scores >70, and only 10 clades had low bootstrap values, grouping sequences that could not be confidently assigned elsewhere (Figure 1B). To gain insight into the relative ages of the initial clades, we used the Evolutionary Placement Algorithm to place *Arabidopsis lyrata* and *Capsella rubella* NLRs in the *A. thaliana* pan-NLRome (Supplemental Figure 1). Of the 65 initial clades, 53 had representative sequences from either or both outgroups (Supplemental Data Set 1). In the initial partition, the largest clade contained 431 sequences, allowing us to construct de novo full-length alignments and clade phylogenies for all clades. A tree of one of the initial clades, Int14015, containing the resistance gene *RPP8*, is representative of observed evolutionary dynamics and is shown in Figure 1C. This tree contains five well-supported subclades that differ in size and internal diversity, as reflected by the very short internal branch lengths in four out of five subclades. The observation that closely related sequences evolve at very different rates is true not only for RPP8, but throughout the NLR family. RPP1, a well characterized NLR that directly interacts with its target ATR1, also has closely related sequences that are largely identical in different ecotypes (Figure 1D). In fact, all clades with longer branches, i.e. higher amino acid divergence, have closely related clades with paralogous genes that show very little variation between ecotypes. These observations are consistent with the notion that closely related NLR genes are experiencing different selection pressures (Ding et al., 2007).

We iteratively refined the initial clades by splitting them into two or more subclades and repeating the alignment and phylogeny generation steps. We prioritized cutting long, well supported internal branches, and therefore tended to preserve both rapidly evolving and low variability subclades (see Methods). After two iterations, the NLRs fell into 223 non-singleton and 14 singleton clades. The distribution of clade representatives across all ecotypes is summarized in Supplemental Data Set 2. This NLRome partition is somewhat more conservative than the OrthoMCL-based analysis, which produced 464 orthogroups and 1,663 singletons (Van de Weyer et al., 2019). In our final clade assignments, 83% of all clades contained no more than one gene for all represented ecotypes, thus approximating allelic series. Over 90% of all NLRs fell into clades of 20 or more genes, allowing sampling for sequence diversity analysis. Only six large clades that ranged in size from 73 to 323 sequences contained multiple genes for 10 or more ecotypes and could not be split further due to the lack of long internal branches with strong support (Supplemental Data Set 2). The large clades contained RPP1, RPP4/5, RPP39, and RPP8, suggesting that interallelic exchange complicated the phylogeny and prevented separation into allelic series. Taken together, our analyses suggest that pan-genomic NLR repertoires can be clustered into near-allelic series using phylogenetic approaches.

### Sequence analysis of the NLRome clades identifies hvNLRs

**Figure 2:**
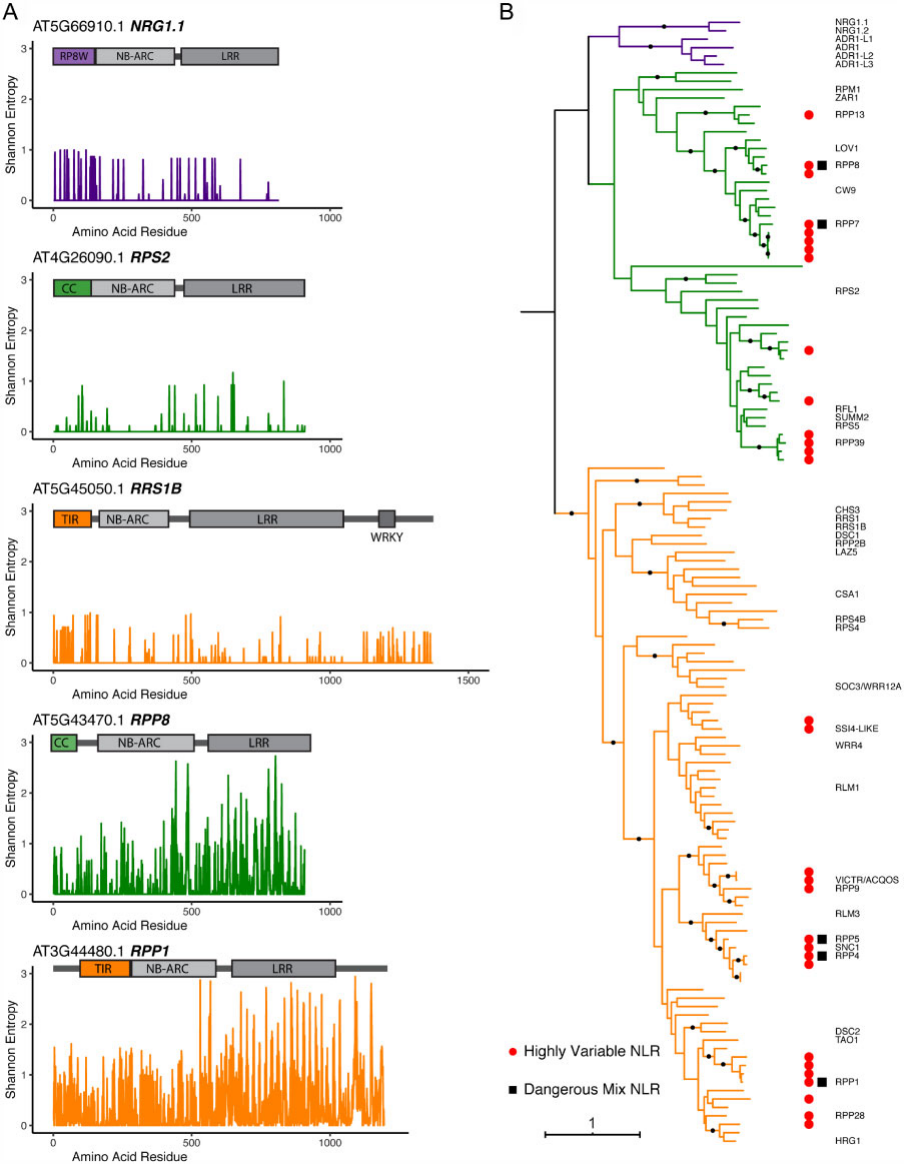
Identification and phylogenetic distribution of hvNLRs. A, Domain diagrams and Shannon entropy plots of clade alignments containing known NLRs from ancient helper (NRG1.1), guard (RPS2), integrated decoy (RRS1B), and direct recognition (RPP1) functional groups. It is not presently known whether RPP8 is a direct recognition NLR. B, Phylogenetic distributions of NLRs of the reference ecotype, Col-0, indicating positions of known genes and showing the locations of hvNLRs and autoimmune Dangerous Mix NLRs. Ninety-nine percent or better bootstrap values are shown as dots; branch length represents the number of substitutions per site.

NLR genes encode immune receptors that provide protection during pathogen infection. Their highly variable regions are expected to contain the specificity-determining residues. We used Shannon entropy as a sensitive and robust measure of amino acid diversity. Entropy is zero at positions that are invariant, and it reaches a theoretical maximum of log_2_20 or ∼4.32 when all 20 amino acids are present in equal ratios; a position with two variant amino acids present at equal ratios produces a value of 1 bit. A Shannon entropy plot thus represents a fingerprint of sequence diversity encoded in the alignment (Figure 2A).

Several functional classes of NLRs produced entropy plots with limited diversity. The ancient helper RNL NRG1.1, the indirect recognition CNL RPS2, and the integrated-domain TNL RRS1B produced entropy plots in which entropy never exceeded 1 bit. The low sequence variability in these clades is consistent with their conserved functions. By contrast, 30 NLR genes in the reference ecotype Col-0, including 14 CNL genes and 16 TNL genes belonged to clades whose alignments repeatedly scored above 1.5 bits and revealed a series of periodic spikes in the LRR region. Among these genes were the known direct recognition proteins from the RPP13 and RPP1 clades. Using Shannon entropy as a metric, we defined hvNLRs as those with 10 or more positions exceeding 1.5 bit cutoff (see Supplemental Figure 2 for the relevant distribution). No protein known to indirectly recognize pathogen effector was found among hvNLRs, and all known direct binders were detected among hvNLRs (Figure 2B). When we ran Shannon entropy analyses on the previously identified NLR orthogroups (Van de Weyer et al., 2019), we only detected 15 hvNLRs, five of which did not overlap with our phylogeny-based analyses (three slightly below 1.5 bits cutoff and two not supported as true orthogroups by phylogeny). This suggests that phylogeny-based orthogroup assignment is a better option for preserving and detecting hvNLR clades. We predict that phylogeny-based NLR clade analysis combined with Shannon entropy can be applied to nonmodel plants to computationally separate candidate direct binders from other NLRs based on their sequence diversity.

### hvNLRs are distributed throughout the TNL and CNL clades

We observed that hvNLRs were distributed over the NLR tree of the reference accession Col-0 with representatives in both TNL and CNL major clades. Within both major clades, there were multiple hvNLR genes right next to conserved paralogs that did not show excess diversity. This is consistent with our prior observation that NLR subclades with long branches have close paralogs with limited subclade diversity. Recent duplications of hvNLRs have produced local hvNLR clusters such as those near *RPP7*, *RPP39*, *RPP4/5*, and *RPP1*. NLRs found in phylogenetic proximity often also cluster physically on the Arabidopsis chromosomes (Supplemental Figure 3). Nonetheless, genomic clustering with close paralogs is not required for an NLR to become highly variable, as shown by *RPP9*, *RPP13*, and *RPP28*. Also, presence in a physical cluster does not force a gene to become an hvNLR, as shown by *RLM3* in the *RPP4/5* genomic cluster and *CW9* in the *RPP7* genomic cluster. Thus, it appears that the copy number variation observed in the clusters is an independent process that helps create material for NLR evolution, but the generation of hvNLRs can proceed outside of genomic clusters.

The physical proximity and phylogenetic relationships of hvNLRs and their closely related low variability paralogs suggest that rapid switches in selective pressure were involved in generating the apparent diversity. Since the selection of an NLR is likely to correlate with its function, we located the known guardian NLRs within the phylogeny. Since these NLRs are expected to maintain binding sites for conserved plant proteins, we expected them to show low entropy scores. As we have already seen for RPS2, other known guardian NLRs including RPM1, RPS5, and ZAR1 all showed low variability. However, they did not form a separate clade within the phylogeny; instead, they were interspersed by hvNLRs. This phylogenetic arrangement, together with the excess of both copy number variation and amino acid diversity in the hvNLRs, argues for a mechanism where hvNLRs mostly act in direct recognition mode but are infrequently able to generate indirect recognition alleles that are preserved due to their competitive advantage.

### hvNLRs contain the known NLR autoimmune loci

Generating diverse receptors in the immune system carries with it a cost of autoimmune recognition. In the known Dangerous Mix gene pairs, at least one and sometimes both causative alleles are NLRs (Chae et al., 2014). If our prediction that hvNLRs are sources of novel direct binding is correct, we would expect to find a strong overlap between hvNLRs and Dangerous Mix NLRs. Indeed, hvNLR clades contain all the known NLR Dangerous Mix genes including *RPP7*, *RPP8*, *RPP4/5*, and *RPP1*. We suspect that in the future, more Dangerous Mix NLRs will be found that will map to other hvNLR loci. This finding also suggests that targeted resequencing of NLRs in crop species could help identify loci responsible for hybrid necrosis phenotypes, which are a frequent impediment to breeding.

### Highly variable residues cluster on the surfaces of LRR domains of hvNLRs

The LRR domains are known to encode the recognition specificities of plant NLRs. First, we wanted to know whether highly variable residues occur predominantly in the LRR domain. This was indeed the case for all 30 hvNLRs examined (Table 1). We noticed, however, that regions in the NB-ARC domain also had high entropy scores in multiple NLRs (RPP1 and RPP8 in Figure 2A). This suggests that a limited number of residues in the NB-ARC domain could participate in target binding in these receptors. Alternatively, these could compensate for changes in the LRR in order to maintain the off state in the absence of the ligand. Many TNLs have post-LRR domains that lack the characteristic LRR pattern of residues yet are predicted to be folded and form a contiguous structure with the preceding repeats (Van Ghelder and Esmenjaud, 2016). We observed that the post-LRR domains also often contained residues with high entropy scores (RPP1 in Figure 2A). Together, these data suggest that the LRR carries the majority of binding residues, while NB-ARC and post-LRR domains can also participate in ligand binding.

**Table 1:** Number and locations of highly variable residues in hvNLR receptors

Gene Name	Type	preNB		NB-ARC		Linker		LRR		postLRR	
		No. of hv aa	Percentage of total aa	No. of hv aa	Percentage of total aa	No. of hv aa	Percentage of total aa	No. of hv aa	Percentage of total aa	No. of hv aa	Percentage of total aa
RPP9	TIR	0	0	0	0	0	0	23	5.8	11	5.3
RPP7	CC	0	0	0	0	1	1.5	34	6.1	0	0
AT1G58807.1	CC	1	0.6	0	0	0	0	29	6.7	1	3.4
AT1G58848.1	CC	1	0.6	0	0	0	0	37	7.2	0	0
AT1G59124.1	CC	1	0.6	0	0	0	0	17	5.6	0	0
AT1G59218.1	CC	1	0.6	0	0	0	0	36	7.1	1	7.7
AT1G61180.1	CC	2	1.3	7	2.1	0	0	35	9.9	0	0
RPP39	CC	2	1.3	4	1.2	0	0	36	8.7	1	2.7
AT1G61300.1	CC	2	4.8	7	2.1	0	0	35	9.9	0	0
AT1G61310.1	CC	2	1.3	7	2	0	0	35	9.9	0	0
AT1G62630.1	CC	0	0	4	1.2	0	0	23	7	2	4
AT1G69550.1	TIR	0	0	2	0.6	2	2.4	58	9.8	1	0.6
RPP28	TIR	1	0.4	0	0	1	3	18	3.7	5	3.4
AT3G44400.1	TIR	2	0.9	4	1.3	3	5.1	22	8.1	18	11.6
RPP1	TIR	3	1.1	6	1.9	3	5.1	35	9.6	17	9.1
AT3G44630.1	TIR	3	1.1	6	1.8	3	5.1	35	9.5	15	8.3
AT3G44670.1	TIR	4	1.5	4	1.3	3	5.1	30	8.9	19	8
RPP13	CC	0	0	0	0	1	2.5	34	11.6	0	0
RPP4	TIR	3	1.6	5	1.7	5	8.3	45	8.4	1	1.6
SNC1	TIR	6	3.2	5	1.7	5	8.5	34	5.5	5	3.6
AT4G16920.1	TIR	7	3.8	5	1.7	5	8.5	51	8.3	3	2.1
RPP5	TIR	7	3.7	5	1.7	5	8.5	41	6.6	8	2.9
AT5G38350.1	TIR	0	0	3	0.9	1	1.7	13	4.6	6	4.1
SSI4-LIKE	TIR	0	0	3	1	0	0	21	6.6	4	2.2
AT5G41750.1	TIR	0	0	2	0.6	1	1.9	19	6	2	1
RPP8	CC	0	0	10	2.9	0	0	19	5	0	0
AT5G43740.1	CC	0	0	2	0.6	2	4.1	27	8.2	0	0
AT5G46510.1	TIR	1	0.5	1	0.3	1	0.9	7	2.3	6	1.4
VICTR/ACQOS	TIR	1	0.5	1	0.3	1	0.9	7	2.3	6	2.3
AT5G48620.1	CC	0	0	10	2.9	0	0	19	5.3	0	0

The number of residues in clade alignment for each hvNLR with Shannon entropy values of at least 1.5 bits (counted by domain) is shown. The majority of highly variable residues were found in the LRR domain.

**Figure 3:**
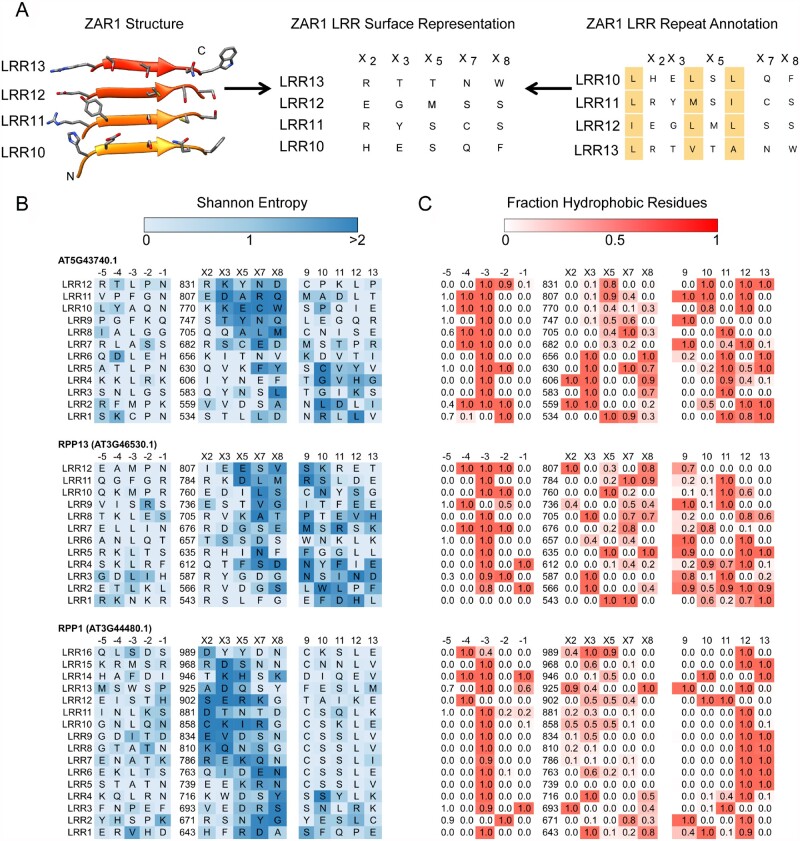
2D representations of LRR surfaces allow comparisons of predicted NLR binding sites to be made in the absence of experimental structures. A, Beta-sheet on the concave side of ZAR1 LRR domain shows a regular array of surface-exposed residues that correspond to the variable positions in the LxxLxLxx LRR motif (left). Single-letter amino acid representation of the observed array (center). Identical representation is obtained from LRR repeat annotation by arranging the rows from bottom to top and hiding the columns containing conserved leucines (right). B, Shannon entropy scores and amino acid residues of three representative Col-0 hvNLRs mapped onto the 2D surface representation, including five additional amino acids on either side of the core repeat unit. C, Percentages of hydrophobic residues in the alignments of the same three proteins.

If the high entropy residues do indeed make up the target binding sites, we would expect to find them in one or two clusters on the receptor surfaces and to include exposed hydrophobic residues. LRR domains fold in a predictable manner that buries the conserved leucines and exposes the variable residues on the protein surface; this allows us to skip structure prediction and to approximate LRR surfaces based on repeat annotation. The concave side of LRR domains contains a beta-sheet with a regular array of surface-exposed residues, and it can be represented as a table with one line per repeat unit and the columns corresponding to variable positions in the canonical Lx_2_x_3_Lx_5_Lx_6_x_7_ repeat. In the case of ZAR1, the first plant NLR whose structure was elucidated, such matrix representation based on repeat annotation perfectly matches the one that is based on the experimental structure (Figure 3A).

In order to test whether entropy analysis can predict NLR binding sites, we annotated LRRs for each hvNLR gene in Col-0 and mapped entropy scores onto this representation. This analysis revealed that in all the hvNLRs, the periodic spikes in entropy signal over the LRR likely correspond to one or two surface clusters in the NLR protein (Figure 3B for three representative examples, Supplemental Data Set 3 for all Col-0 hvNLRs). In the first example, AT5G43740, the strongest variability signal is found in LRRs 8 through 12 and positions 3, 5, 7, and 8 of the repeat. Additional high entropy signal comes from LRR1 through LRR5 positions 8 and 10. In RPP13, the positions C-terminal to the predicted beta sheet appear to play an important role in determining binding specificity. Unlike AT5G43740, highly variable residues in positions 8, 9, and 10 of the repeats appear throughout the annotated LRR region, while all residues in positions 2 and 3 are conserved. We therefore predict that in RPP13, loops that follow the beta strands play a key role in determining substrate specificity. Our prediction that specificity determinants of RPP13 stretch between LRR1 and LRR12 are in agreement with the large experimentally identified specificity-determining region in RPP13 (Rentel et al., 2008, and see Figure 5 below).

RPP1 is a well-studied example of a direct recognition NLR where multiple alleles have different recognition profiles of the effector ATR1 of the downy mildew pathogen *Hyaloperonospora arabidopsidis* (Rehmany et al., 2005). In RPP1, we observed a large number of contiguous residues that likely contribute to binding specificity stretching from LRR1 to LRR15. Highly variable residues are concentrated in positions 5, 7, and 8 at the beginning of the domain but shift toward the start of the beta strands in the later repeat units, with residues 2, 3, and 5 lighting up uniformly in LRR7–LRR15. Rather unusually, we also observed some variable residues in the −1 and −2 positions. We conclude that in RPP1 (and in AT5G43740) the targets likely bind through the middle of the horseshoe LRR shape rather than on one side of it, as in the case of RPP13. The high-entropy residues in RPP1 contain the amino acids previously shown to extend recognition specificity of the RPP1 allele NdA towards ATR1-Maks9 (Krasileva, 2011) and those that directly interact with ATR1 in the cryo-EM structure (Ma et al., 2020; see Figure 6 below).

To further investigate whether the identified highly variable surfaces indeed represent target-binding sites, we surveyed these regions of high diversity for the presence of exposed hydrophobic residues, which are commonly found at the centers of protein–protein binding sites (Figure 3C). Indeed, in every case, the highly variable residues included exposed hydrophobic amino acids, often including bulky aromatics such as tryptophan and phenylalanine. We also tested whether the entropy-based predictions agree with the results of positive selection analyses that have been used in the past to identify functionally important residues in NLRs (Kuang et al., 2004). In RPP13, 66% of all high-entropy residues (>1.5 bits) were under positive selection according to phylogenetic analysis by maximum likelihood (PAML) Model 8 (Supplemental Figure 4). All of the remaining high-entropy residues fell into regions that contained gaps in the alignment and could not be analyzed by PAML. Thus, the results of the entropy analyses of hvNLR surfaces are consistent with the results of the widely accepted molecular evolution analyses performed on the underlying nucleotide sequences.

### NLR-binding sites are largely similar across the NLRome

**Figure 4:**
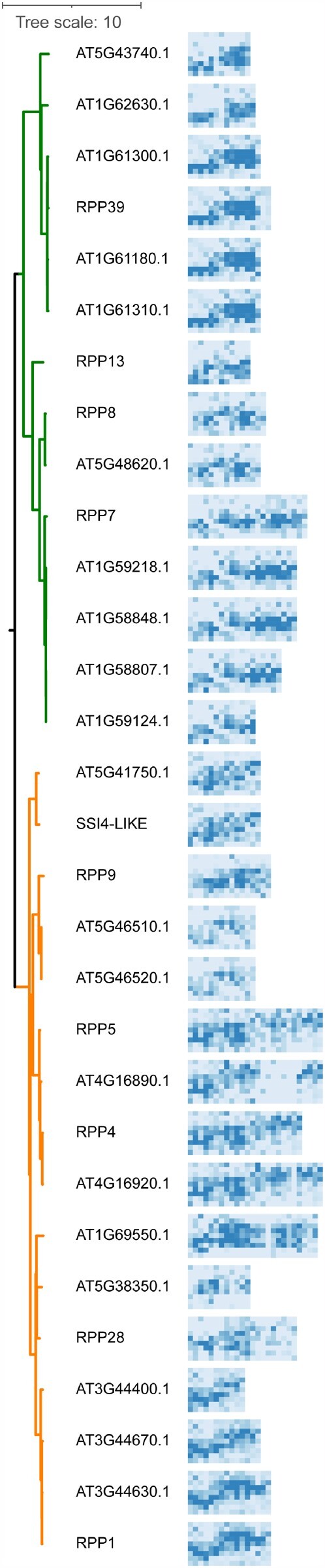
2D representations of Col-0 hvNLR LRR surfaces in the context of the Col-0 NLR tree. The 2D binding site representations are those in Figure 3 and Supplemental Data Set 3 situated horizontally and trimmed to include positions −2, −1, 2, 3, 5, 7, 8, 10, and 11 of each repeat unit. For each cartoon, the −2 position of LRR1 is in the top left corner and the position 11 of the last LRR is in the bottom right corner. The tree on the left is a subset of the Col-0 NLR tree from Figure 2B with only the hvNLR leaves shown.

We next examined how the placement of the highly variable residues and the predicted ligand binding site evolved across the NLR phylogeny (Figure 4). Overall, closely related paralogs shared a similar binding site location, and most variation was apparent between CNLs and TNLs. We observed that the clustering of highly variable residues was largely similar across CNLs, with most sites clustering together in C-terminal repeats and most variability introduced by the repeat number variation. In TNLs, highly variable sites were more dispersed across the LRRs, and the predicted binding site was stretched across NLRs with a larger number of repeats. Across both TNLs and CNLs, the N-termini of LRRs 1–4 were invariable: this region is in contact with the invariable part of the NB-ARC domain and might be important for regulating NLR activation.

### The ZAR1-RKS1 binding site overlaps with the binding site of RPP13 predicted by entropy-based analysis

**Figure 5:**
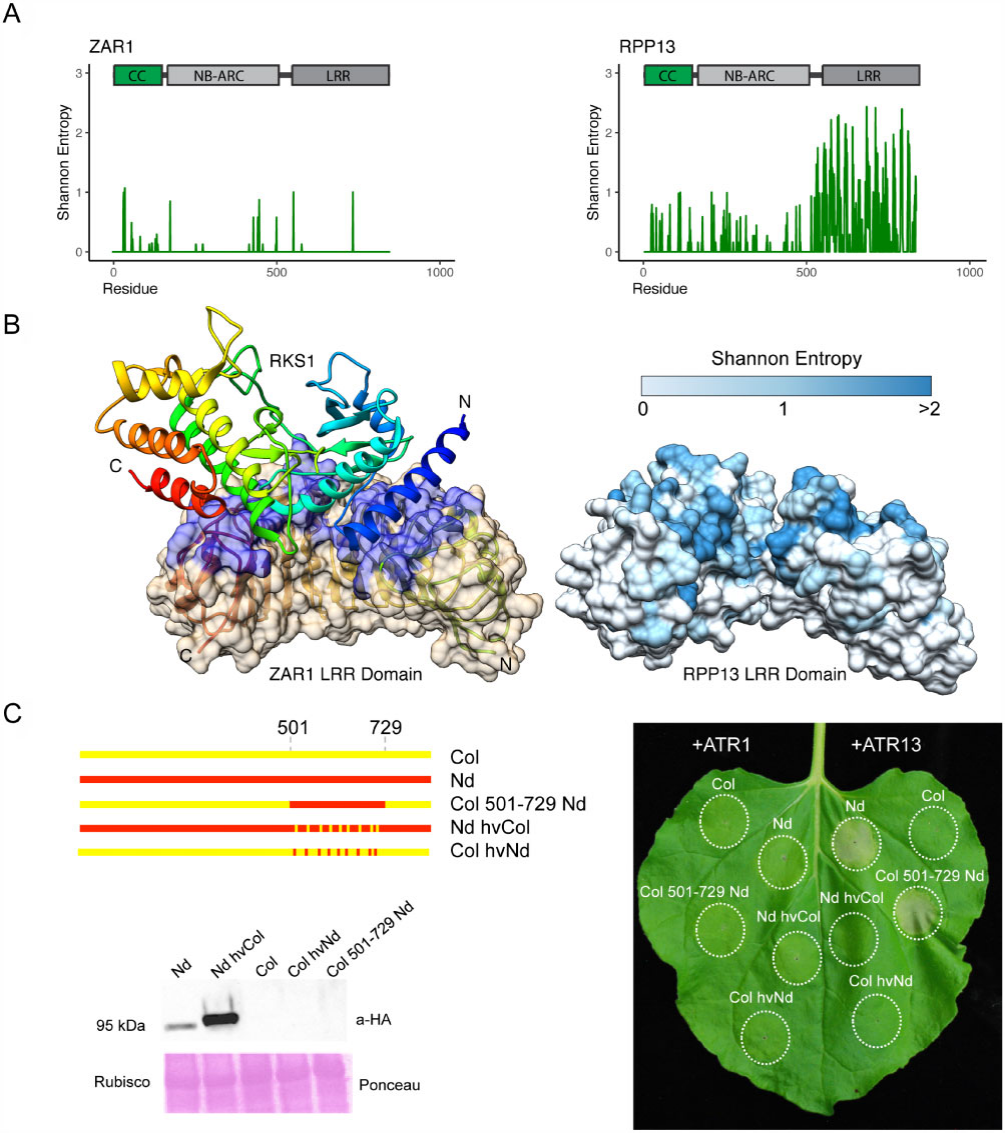
Highly variable residues in RPP13 overlap with the observed ZAR1-RKS1 binding site and are required for ATR13 recognition. A, Shannon entropy plots and domain diagrams for ZAR1, an indirect recognition CNL, and RPP13, a related hvNLR. B, Cryo-EM structure of RKS1 bound to ZAR1 (CC and NB-ARC domains omitted for clarity; PDB ID: 6J5W). RKS1 shown as a secondary structure diagram with rainbow coloring from blue (N-terminus) to red (C-terminus), ZAR1 LRR as a secondary structure diagram and transparent surface with RKS1 contact residues colored blue. RPP13 LRR domain homology model with surface oriented as in ZAR1 and colored by Shannon Entropy of the RPP13 clade alignment from low (light blue) to high entropy (dark blue). C, Chimeric constructs of RPP13 regions 501–729 containing highly variable LRR repeats. The constructs were designed by targeting amino acids with Shannon entropy >1.5 bits and functionally tested by Agrobacterium-mediated transient expression assays in *N. benthamiana* together with cognate ATR13d41-Emco5 effector or ATR1d51-Emoy2-negative control at the final OD_600_ of 0.6 with constructs mixed in equal ratio. The image was taken at 3 days post infiltration. Each construct was tested on 14 leaves and showed consistent presence/absence of HR on all leaves. Immunoblotting showed stable expression of both functional and mutated RPP13-Nd variants. No RPP13-Col variants could be detected despite having an intact HA tag similar to what has been reported previously (Rentel et al., 2008).

Arabidopsis ZAR1 is an indirect-recognition NLR and the first one with an elucidated structure. In our phylogeny, its closest hvNLR is RPP13 (Figure 2B). While the ZAR1 entropy plot lacked high-entropy residues, we wanted to compare the known footprint of RKS1, the ZAR1 binding partner, with the positions of highly variable residues in RPP13 (Figure 5A). Unusually for hvNLRs, highly variable residues of RPP13 cluster on the C-terminal side of the repeats, with positions 7–10 of the repeat units showing the highest diversity (Figure 3). Surprisingly, the similarly positioned residues in ZAR1 are used to bind its stable complex partner, RKS1 (Figure 5B). This finding is consistent with the notion that ZAR1 and RPP13 emerged from an hvNLR common ancestor that had a binding site similar to that observed in ZAR1 and predicted in RPP13.

### High-entropy residues in RPP13 are required for recognition of ATR13

To experimentally test our prediction, we created synthetic RPP13 constructs and transiently expressed them in *Nicotiana benthamiana* together with the ATR13 d49 Emco5 allele, which is recognized by RPP13-Nd but not RPP13-Col. We used another effector, ATR1 d51 Emoy2, which is not recognized by either RPP13 variant, as a negative control. RPP13-Col containing the 509–729 amino acid region from the Nd allele showed a gain of ATR13 recognition, which is consistent with our prediction (Figure 5C). Similarly, swapping 21 amino acids with Shannon entropy >1.5 bit from Nd to Col created a loss-of-function allele, despite stable protein expression, confirming the functional requirement for highly variable residues (Figure 5C;  Supplemental File 3). However, the same 21 amino acids transferred from RPP13-Nd to RPP13-Col were not sufficient for a gain of recognition, suggesting that residues with lower entropy scores also participate in target binding. (Neither functional nor nonfunctional RPP13-Col variants could be observed by immunoblotting, as reported previously (Rentel et al., 2008).)

### The majority of RPP1 target-binding site residues show high sequence variability

**Figure 6:**
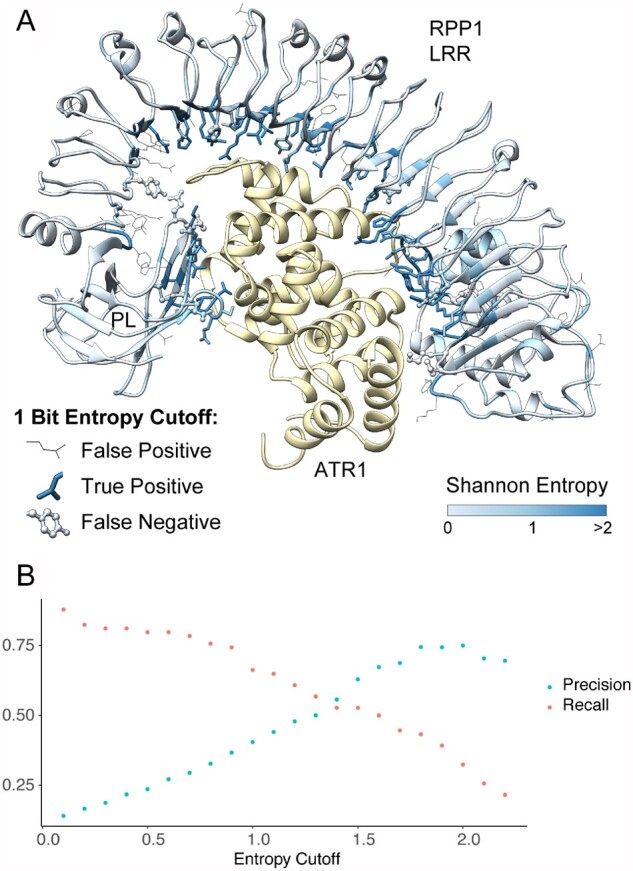
RPP1 contact residues show high sequence diversity. A, Structure of RPP1 LRR-ATR1 complex (PDB ID: 7CRB) colored by entropy scores, with contact residues shown as sticks for predicted true positives and ball and stick for false negatives using a 1 bit entropy cutoff. False positive predictions at the same cutoff are represented as wire. B, Precision and recall for the prediction of RPP1-ATR1 binding site residues based on the choice of entropy cutoff.

While this manuscript was in review, the cryo-EM structure of RPP1 bound to ATR1 was published (Ma et al., 2020), allowing us to directly evaluate the accuracy of our binding site predictions. The majority of binding residues had entropy values above one bit (Figure 6A). Both precision (fraction of positives among all predictions) and recall (fraction of positives recovered) varied with the entropy cutoff chosen. Maximal recall was achieved at a cutoff of 0.8 bit, and precision improved up to a cutoff of 1.8 bits. Thus, cutoff values in this range are likely to be useful, with higher cutoffs achieving greater accuracy at the cost of missing a greater number of true positives (Figure 6B). Our empirical 1.5 bit cutoff used to define hvNLR clades is therefore a conservative one. It is also important to note that sequence-based analyses predicted a number of RPP1-binding residues past the LRR domain (Table 1); the structure revealed that these residues form a contiguous surface on the post-LRR domain that is characteristic of a number of TNL receptors.

### hvNLRs show a similar phylogenetic distribution in *B. distachyon*

To test whether our methods and findings are applicable beyond *A. thaliana*, we performed a similar analysis on 54 lines of *B. distachyon*, a model grass species. The automatic short-read assembly and annotation pipeline used to generate the Brachypodium data is less reliable than the targeted resequencing approach used to generate Arabidopsis pan-NLRome. Specifically, only 45% of hvNLRs present in reference strain Bd-21 were recovered in the assembly control. Nonetheless, the overall picture that emerged from the analysis of Brachypodium NLR clades is similar to that of Arabidopsis. After splitting the overall Brachypodium NLR tree into 91 initial clades, we performed four rounds of clade refinement to arrive at a final clade partition with 433 subclades. Of these, 28 produced alignments that fulfilled the hvNLR criteria. Altogether, 40 hvNLRs in the reference accession Bd21 were identified as hvNLRs.

**Figure 7:**
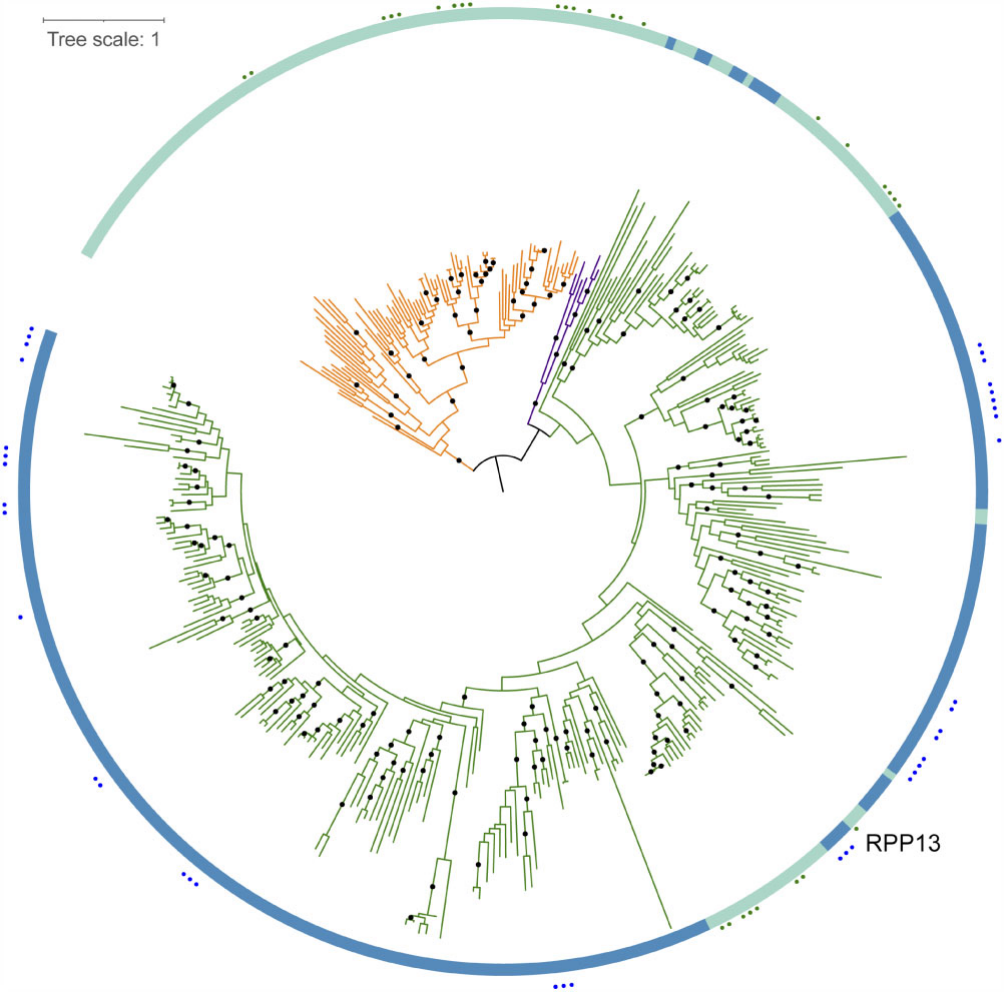
Dispersed distribution of hvNLRs in a joint phylogeny of Brachypodium Bd21 (blue ribbon) and Arabidopsis Col-0 (green ribbon). The Arabidopsis hvNLR clades (green dots) and Brachypodium hvNLRs (blue dots) do not cluster except for the RPP13 CNL clades. The tree is rooted arbitrarily on a branch connecting TNL clade (orange branches) and non-TNL clades (RNL branches are shown in purple and CNL branches are shown in green). Ninety-nine percent or better bootstrap values are shown as dots; branch length represents the number of substitutions per site.

Similar to *A. thaliana*, Brachypodium hvNLRs were distributed throughout the phylogeny, including in the highly expanded monocot-specific CNL clade. Here too, hvNLRs had sister clades that showed little amino-acid diversity. Importantly, when we constructed the joint tree for Col-0 and Bd21 reference NLRomes, the only hvNLRs from the two species that appeared close together belonged to the RPP13-like clades (Figure 7). This highlights the importance of sequencing the pan-NLRomes of plants of interest, as the identification of hvNLRs is unlikely to be transferable except for closely related species.

## Discussion

Even before the first NLR structure or the extensive sequence datasets were available, Michelmore and Meyers predicted that hypervariable amino acid positions in the NLRs would map to the concave surface of the LRR domain based on the signatures of positive selection in a number of selected examples (Michelmore and Meyers, 1998). They generalized that this might be true for all NLRs. This model was challenged by the discovery of indirect recognition and of strongly conserved NLRs. Our analysis proposes a powerful methodology to study NLR-omes, predicts NLR mode of action through sequence analysis, and reconciles the evolution of direct recognition NLRs (under diversifying selection) and indirect recognition NLRs (under purifying or balancing selection).

In this study, we observed that hvNLRs account for the known direct recognition NLRs and for autoimmune NLRs. We also observed that the hvNLRs have close paralogs with little allelic diversity that include the known indirect recognition NLRs. Based on this observation, we propose that indirect recognition NLRs are a functional byproduct of hvNLR evolution, providing an important update of the birth-and-death model (Michelmore and Meyers, 1998). Our analyses suggest that in a given species, diversity generation occurs in a limited subset of NLR genes, creating a wide recognition potential, including binding to endogenous plant proteins. When recognition of endogenous proteins is beneficial, such as under perturbations by the pathogen, the NLR evolves into indirect recognition and begins to experience different selective forces.

The resolution and sensitivity of our analyses became possible when we adopted two key approaches: identifying orthologous groups of NLR receptors by phylogeny in place of commonly used distance metrics; and using simpler Shannon entropy measure of diversity in place of more complex evolutionary models. Separating rapidly evolving protein families into meaningful clades or groups for downstream analyses is a common challenge. In the NLR family of plant immune receptors, this process is further complicated by ongoing information flow between close paralogs through recombination and gene conversion (Kuang et al., 2004). Phylogeny-based analyses are considered to be more accurate than distance-based methods for similar problems such as classifying Human Immunodeficiency Virus isolates (Pineda-Peña et al., 2013). Our phylogeny-based partition of NLR immune receptors into clades improved on the published OrthoMCL-based partition by producing more encompassing clades and (in particular) fewer singletons. OrthoMCL is a distance-based algorithm that was originally developed to separate members of different protein families rapidly; it uses a single parameter to determine the rate of convergence (Li et al., 2003). This makes its use to partition the pan-NLRome problematic, because closely related NLRs are known to experience vastly different selection pressures and thus are expected to contain very different amounts of allelic diversity (Kuang et al., 2004; Bakker et al., 2006). The specific danger for hvNLR identification is that highly variable clades will be split, losing the relevant signal. This is indeed what we observed, as the OrthoMCL-based analysis identified only one out of three hvNLRs and missed key sources of new NLR specificity such as the RPP1 cluster, which was split into small orthogroups. The drawback of the phylogeny-based approach is that it is not yet fully automated; however, we are hopeful that phylogeny-aware algorithms will emerge that will fill this gap. One alternate approach that would simplify the analysis would be to replace the initial clade assignment with iterative matching of NLR sequences against a set of inferred ancestral NLR models (Shao et al., 2016).

It is well established that closely related NLRs experience different modes of selection (Kuang et al., 2004; Ding et al., 2007; Wang et al., 2011). By expanding this observation to the pan-NLRome and combining it with the wealth of characterized NLRs in Arabidopsis, we were able to decipher a larger evolutionary pattern where hvNLRs act as sources of new specificities and encompass the known direct-recognition NLRs. Their diversification, while advantageous to the plant, comes at a cost. All known Dangerous Mix NLR genes that can trigger autoimmune recognition belong to hvNLR clades. Thus, the generation of novel specificities goes hand in hand with the potential for self-recognition and auto-immunity. We also propose that during their continuous evolution, hvNLRs can generate indirect-recognition NLRs at a low frequency. Because indirect recognition usually tracks a conserved effector activity, it is more robust than direct recognition of the effector surface. Duplication of such successful variants might then be favored due to the increased fitness of the progeny where one copy could eventually be preserved while the other could continue to generate novel specificities (Kondrashov et al., 2002). The latter inference is consistent with our observation that ZAR1, an indirect-recognition NLR, binds to its stable complex partner RKS1 through the same surface on the LRR that contains highly variable residues in RPP13, its closest hvNLR.

When we applied Shannon entropy analysis to the NLR clades, only a subset of clades gave strong signals; these clades included known direct recognition NLRs and autoimmune NLRs. When we looked at the distribution of high-entropy amino acids in the 30 hvNLRs of Arabidopsis reference strain Col-0, we found that these residues commonly clustered on the predicted surfaces of LRR domains. This observation is consistent with the finding that binding specificities are largely encoded in the LRR domains, as supported by multiple genetic and biochemical studies (Ellis et al., 2007; Krasileva et al., 2010), as well as the prediction (by evolutionary studies) that amino acid residues under positive selection are located within LRRs (Kuang et al., 2004; Rose et al., 2004; Wang et al., 2011). When we carried out a positive selection analysis on the RPP13 clade, we found that the majority of residues with entropy >1.5 bits were under positive selection. The only exceptions were residues that could not be analyzed for positive selection due to the presence of gaps in the relevant alignment columns. Shannon entropy calculation does not count gap characters. Instead, it works without making complex assumptions about the data and is therefore much faster computationally.

In our analysis, we went a step further to predict binding sites in hvNLRs directly from pan-NLRome sequence data. The identified binding sites are large. This is likely in due (in part) to the concave shape of the LRR scaffold, which can place many of the beta strands in contact with a relatively small target. Comparisons of antibody sequence-based predictions with experimental structures showed that the predictions correctly recover ∼80% of residues that do contact the antigen, while also producing many false-positives (<50% precision; Kunik et al., 2012). Unlike the antibody, where the binding determinants are present on loops away from the core of the structure, in the LRR, many predicted binding residues fall within the beta sheet located on the concave side of the domain. This suggests that the accuracy of the prediction might be higher in this system due to stronger structural constraints. Additional highly variable residues were located in post-LRR domains and in specific sites within NB-ARC, suggesting their involvement in substrate binding, or in case of NB-ARC of a compensatory mechanism to maintain self-inhibition in the absence of the ligand. Further mutational and structural experiments in well-established NLR-effector systems would be needed to test the accuracy of these predictions and to help refine them.

Identification of the immense allelic diversity across hvNLRs argues that plant immunity is not far in its allele-generating potential from the most well-known adaptive immune systems. Indeed, LRRs are deployed in the adaptive immune systems of early-diverging vertebrates, demonstrating that their modularity is sufficient for the generation of binding to any foreign molecule (Han et al., 2008; Das et al., 2013). In the case of plants, enormous diversity is generated at the population level rather than within a single organism, and therefore, defending against new pathogens is a community effort. The identification of specific genes within crop species capable of such diversity generation and their deployment in protein engineering efforts could provide valuable material for plant health.

We conclude that phylogenetic analysis of pan-NLRomes combined with sequence diversity analysis can rapidly classify NLRs into functional groups given sequencing information for at least 40–60 diverse samples. We also believe that our method would be generally applicable to the identification of highly variable RLP, such as Cf-9 in tomato (Wulff et al., 2009), and the prediction of binding sites of highly variable extracellular immune receptors. Our method can also predict incompatibility loci, which can be taken into account in breeding new crop varieties. Similar allelic diversity analyses in other nonvertebrate eukaryotes with expanded immune receptor families are needed to test whether the patterns of innate immune receptor evolution we observed are shared across the eukaryotic kingdoms of life.

## Materials and methods

### Phylogenetic analysis

Phylogenetic tree construction for the *A. thaliana* and *B. distachyon* NLRomes and the NLRomes of reference accessions was performed as previously described (Bailey et al., 2018). Briefly, amino acid sequences were searched for the presence of the NB-ARC domain using hmmsearch (Mistry et al., 2013) and the extended NB-ARC Hidden Markov Model (HMM) 13059_2018_1392_MOESM16_ESM.hmm (Bailey et al., 2018), and initial alignment was made on this HMM using the -A option. The resulting alignment was processed with Easel tools (https://github.com/EddyRivasLab/easel) to remove insertions and retain aligned sequences that matched at least 70% of the HMM model. This alignment was used to construct maximum likelihood phylogenetic trees using RAxML software version 8.2.12 (Kozlov et al., 2019; raxml -T 8 -n Raxml.out -f a -x 12345 -p 12345 -# 100 -m PROTCATJTT). The sequences of outgroup species were aligned to the same NB-ARC HMM and placed in the pan-NLRome tree using RAxML Evolutionary Placement Algorithm . The trees were visualized in the Interactive Tree Of Life (iTOL) (Letunic and Bork, 2019).

### Initial clade assignments

The phylogeny was used to separate protein sequences into clades using R scripts prefix_Initial_Assignment.R (hereafter the prefix is either Atha_NLRome or Brachy_NLRome for the two species under analysis). This and other scripts referenced below are available at (https://github.com/krasileva-group/hvNLR). First, for each NB-ARC sequence, a clade 40–500 in size with the strongest bootstrap support was chosen. For sequences that did not belong to clades in this size range, smaller clades were allowed. Second, the resulting set of clades was made nonredundant by excluding all nesting clades. The resulting partitions uniquely assigned the 7,818 *A. thaliana* NLR sequences to 65 clades and 11,488 *B. distachyon* NLR sequences to 91 clades.

### Iterative clade refinement

For each identified clade, full-length protein sequences were aligned using the PRANK algorithm (Löytynoja, 2014), and phylogenetic trees based on full-length alignments were constructed as described above using RAxML. Trees were visualized in iTOL, along with subclade statistics calculated in R, and R scripts were used to produce subclade lists based on the trimmed branches (prefix_Refinement.R). For the first iteration, gappy columns in the full-length alignments were masked (90% cutoff), and later iterations were analyzed without masking gappy columns. Clade refinement was performed as follows: all tree branches longer than 0.3 were cut to form two or more subclades. All branches 0.1 and shorter were retained in the first iteration, and for the branches between 0.1 and 0.3, the decision to cut was made by visually inspecting the tree in iTOL and considering bootstrap support and overlap in ecotypes on either side of a branch. The sequences belonging to the refined subclades were realigned using PRANK, and tree construction repeated. In the following iterations, some branches shorter than 0.1 were cut via tree inspection in iTOL based on bootstrap support and ecotype overlap. The refinement process converged to produce the final assignment of all genes into 237 final clades for *A. thaliana* and 433 clades for *B. distachyon*.

### Identification of hvNLR clades and prediction of binding sites in hvNLRs

We used R scripts (prefix_CladeAnalysis.R) to calculate alignment Shannon entropy scores using the package “entropy.” Alignments that contained 10 or more positions with at least 1.5 bits were considered highly variable. All highly variable clades were examined for the presence of Arabidopsis Col-0 alleles. For these Col-0 alleles, we predicted the LRR coordinates manually and cross-checked these predictions with an LRRpredictor online server (Martin et al., 2020). R script was used to map entropy scores to the predicted concave surface of the LRR domain (Atha_NLRome_GeneEntropy.R). The entropy scores for the individual strands of LRRs (LxxLxLxx) were exported in tabular format. The hydrophobicity scores of these residues were calculated as the percent of hydrophobic residues at a given amino acid position and exported as a second table. The resulting 2D representations of entropy and hydrophobicity of the concave sides were visually examined for clustering of residues that showed both high entropy scores and the presence of hydrophobic residues. Positive selection analysis of the RPP13 clade alignment was carried out in PAML (Yang, 2007).

### Structural analysis of RPP13 homology model, ZAR1 structure, and RPP1 structure

In order to compare the 3D spatial distribution of highly variable residues in RPP13 with the ZAR1-RKS1 binding site, we used phyre2 in one-to-one threading mode to produce a model for RPP13 (Kelley et al., 2015) based on the ZAR1 experimental structure. The alignment had 24% identity over the complete sequences, with 31% identity before and 15% over the LRR domain. Important for the model accuracy, there were only two gaps of seven residues and two gaps of three residues, with several more single-residue gaps in the LRR domain. Thus, it is unlikely that whole repeat units are missing from the model. R script (Atha_NLRome_GeneEntropy.R) was used to produce a Chimera-formatted attribute files to color the model surfaces by entropy scores, and figures were generated in Chimera (Pettersen et al., 2004). The dependence of binding residue prediction recall and precision on the entropy cutoff was determined using a custom R script (RPP1_Precision_Recall.R).

### Constructs

RPP13-Nd and RPP13-Col cDNA without a stop codon fused to C-terminal HA tag in pENTRY/TOPO-D were obtained from the Staskawicz laboratory (Rentel et al., 2008) and were used to generate chimeric and synthetic RPP13 variants. RPP13 501–729 synthetic constructs with highly variable residues (Shannon entropy cutoff >1.5) swapped between Nd and Col (Supplemental Data Set 4) were designed in SnapGene and synthesized as gene fragments by Integrated DNA Technologies. The clones were digested with uniquely cutting restriction enzymes SacI (New England BioLabs) and MslI (New England BioLabs). The chimeric constructs were ligated for 2 h at room temperature with T4-DNA ligase (New England BioLabs) and transformed into electrocompetent *E. coli* Top 10b (Invitrogen). The resulting constructs were introduced into binary vector pMD:npRPP13 (Rentel et al., 2008) using LR clonase II (Invitrogen) and transformed into *Agrobacterium tumefaciens* GV3101(pMP90RK). ATR1 d51 Emoy2 tagged with C-terminal citrine in pEarleyGate103 (Krasileva et al., 2010) and ATR13 d41 Emco5 in p1776 (Rentel et al., 2008) were used for transient transformation.

### Transient expression

*Agrobacterium tumefaciens* strains were grown for 24–48 h at 28°C in Luria–Bertani broth (100 *µ*g/mL, gentamicin 50 *µ*g/mL, kanamycin 25 *µ*g/mL) with constant shaking. After pelleting, the cells were resuspended in induction medium (10 mM MgCl, 10 mM MES, and 150 *μ*M acetosyringone, adjusted to pH 5.6 with KOH), adjusted to a final OD_600_ of 0.6, and induced for 3 h at room temperature. Co-infiltrations were done at a final OD_600_ of 0.6 and contained constructs mixed in a 1:1 ratio. Fully expanded leaves of 4- to 5-week-old *N. benthamiana* plants grown in Supersoil mix #4 supplemented with Miracle Gro Plant Food fertilizer at 24°C under a 16-h light (fluorescent lamps)/8-h dark cycle were infiltrated using a blunt end syringe. After infiltration, the plants were kept at constant light (fluorescent lamps, GE Cat #F405941-ECO) and room temperature. The hypersensitive response reaction was monitored for 4 days, with pictures taken 3 days post infiltration. Two leaf disks (1.5 cm^2^ in diameter) were collected from RPP13/ATR1 co-infiltrations for protein extraction 2 days post infiltration, frozen in liquid nitrogen, and stored at −80°C.

### Protein extraction and immunoblotting

Tissue in a 1.5-mL Eppendorf tube was frozen in liquid nitrogen a ground with a manual drill using a pre-chilled plastic pestle. Total protein was extracted by re-suspending the ground tissue in 2× Laemmli buffer (Bio-Rad, Cat. #1610737) supplemented with fresh β-mercaptoethanol to a final concentration of 5% (by volume), boiling for 5 min, and pelleting the debris for 10 min at 14,000 rpm. Fifteen microliter of each protein sample was separated on a 4%–15% Mini-PROTEAN gel (BioRad) for 1 h at 100 V and transferred onto a nitrocellulose membrane using wet transfer for 1.5 h at 300 mA. The membranes were blocked overnight in 5% milk in Tris-Buffered Saline with 0.05% Tween 20 (TBS-T), incubated for 1 h in rat α-HA-horseradish peroxidase antibody (clone 3F10; Roche, Cat #12013819001) at 1:1,000 dilution in TBS-T, washed once for 15 min and twice for 5 min in TBS-T, and imaged using SuperSignal West Pico PLUS Luminol substrate (Thermo Scientific) inside a Gel Imager (BioRad). Total protein loading was confirmed by staining the membrane in Ponceau S and destaining in 5% acetic acid.

### Accession numbers

Arabidopsis pan-NLRome nucleotide assemblies were downloaded from the 2Blades foundation (http://2blades.org/resources/). Gene annotations were downloaded from GitHub pan-NLRome repository (https://github.com/weigelworld/pan-nlrome/). The gene models that matched assemblies were available for 62 *A. thaliana* accessions (Van de Weyer et al., 2019), and these were processed to extract the amino acid sequences of captured protein-coding genes using bedtools getfasta program (Quinlan, 2014). The reference set of 168 NLR alleles (including splice variants) of the Arabidopsis Col-0 genome was extracted as described before (Sarris et al., 2016). The accession numbers of RPP13 used in the laboratory experiments are RPP13-Nd (AF209732.1) and RPP13-Col (AF209730.1). The PDB accession number of the RPP1 structure used in this study is 7crb. Brachypodium proteomes for 54 lines were downloaded from BrachyPan (https://brachypan.jgi.doe.gov) (Gordon et al., 2017). The R scripts used to analyze project data are available via GitHub (https://github.com/krasileva-group/hvNLR), the complete data set for the project including clade alignements and clade trees is available via Zenodo (DOI: 10.5281/zenodo.3951781), and the clade trees can be viewed in iTOL (http://itol.embl.de/shared/daniilprigozhin).

## Supplemental data

**Supplemental Figure 1.** *A. thaliana* pan-NLRome tree showing initial clades and phylogenetic placements of outgroup sequences from *A. lyrata* and *C. rubella*.

**Supplemental Figure 2.** Distribution of highly variable sites per final clade alignment.

**Supplemental Figure 3.** Comparison of phylogenetic versus physical clustering of Col-0 NLRs.

**Supplemental Figure 4.** Comparison of entropy-based and positive selection-based binding site predictions.

**Supplemental Data Set 1.** Number of NLRs from *A. lyrata* and *C. rubella* in the initial NLR clades.

**Supplemental Data Set 2.** Number of NLRs in the final NLR clades across the 62 *A. thaliana* ecotypes.

**Supplemental Data Set 3.** 2D representations of LRR surfaces of 30 hvNLRs from ecotype Col-0.

**Supplemental Data Set 4.** Nucleotide and amino acid fasta sequences of RPP13 501–729 synthetic constructs that have highly variable residues swapped between Col and Nd allele.

## Supplementary Material

koab013_Supplementary_Data

## Notes

D.M.P. and K.V.K. designed and performed the research and wrote the article.

The author responsible for distribution of materials integral to the findings presented in this article in accordance with the policy described in the Instructions for Authors (https://academic.oup.com/plcell) is: Ksenia V. Krasileva (kseniak@berkeley.edu).
