# Supplementary material for: Analysis of intraspecies diversity reveals a subset of highly variable plant immune receptors and predicts their binding sites
Source: Plant Cell. 2021 Jan 25;33(4):998–1015. doi: 10.1093/plcell/koab013 (PMC8226289; doi:10.1093/plcell/koab013)
Supplement: koab013_Supplementary_Data [file koab013_supplementary_data.zip › tpc.00673.2020-s02.pdf]

Supplemental Data. Prigozhin and Krasileva (2021). Analysis of intraspecies diversity reveals a subset of highly variable plant immune receptors and predicts their binding sites. Plant Cell.

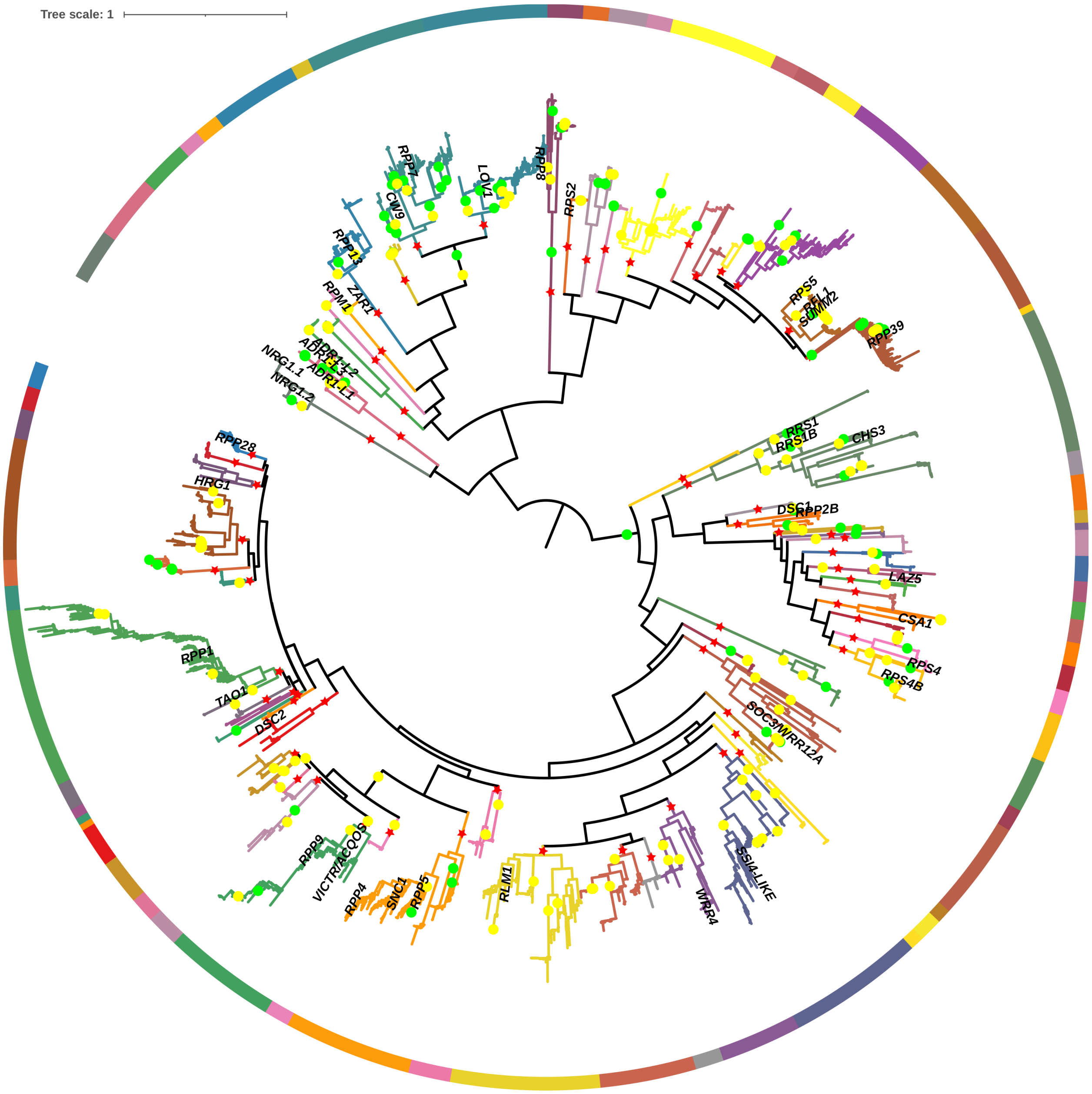

Supplemental Figure 1. *A. thaliana* pan-NLRome tree showing initial clades and phylogenetic placements of outgroup sequences from *A. lyrata* and *C. rubella.*
(Supports Figure 1).
Maximum likelihood tree for 7,818 Arabidopsis NB-ARC sequences rooted on a branch connecting TNL and non-TNL clades. Initial clades are shown as branch and stripe color and have red stars at the clade-defining branches. Phylogenetic placement of *A. lyrata* and *C. rubella* NLRs are shown as yellow and green dots, respectively. Branch length represent the number of substitutions per site.

Supplemental Data. Prigozhin and Krasileva (2021). Analysis of intraspecies diversity reveals a subset of highly variable plant immune receptors and predicts their binding sites. Plant Cell.

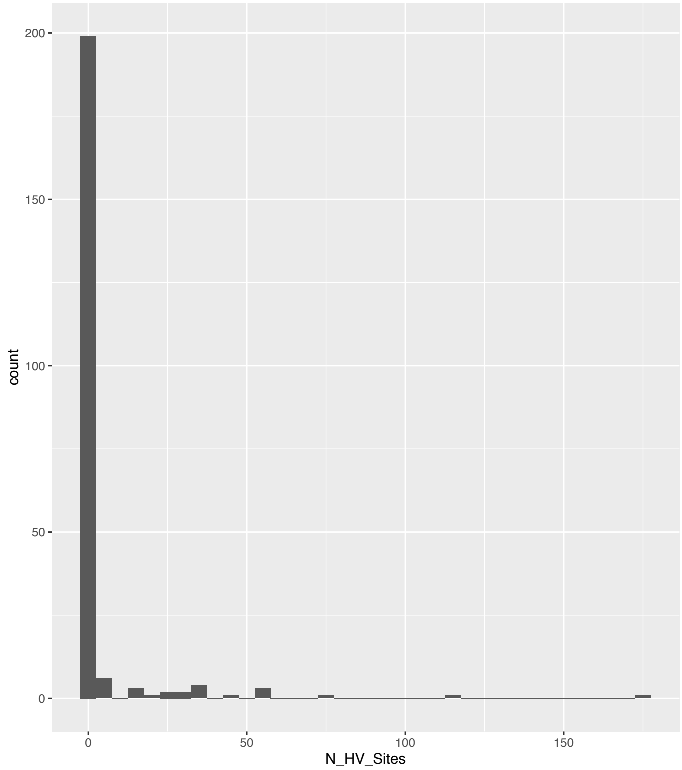

Supplemental Figure 2. Distribution of highly variable sites in alignments of refined clades. (Supports Figure 2)
We calculated the number of amino acid positions with entropy scores exceeding 1 bit in the alignments of the refined clades (N_HV_Sites). The distribution of these counts is shown as a histogram with the bin size of 5.

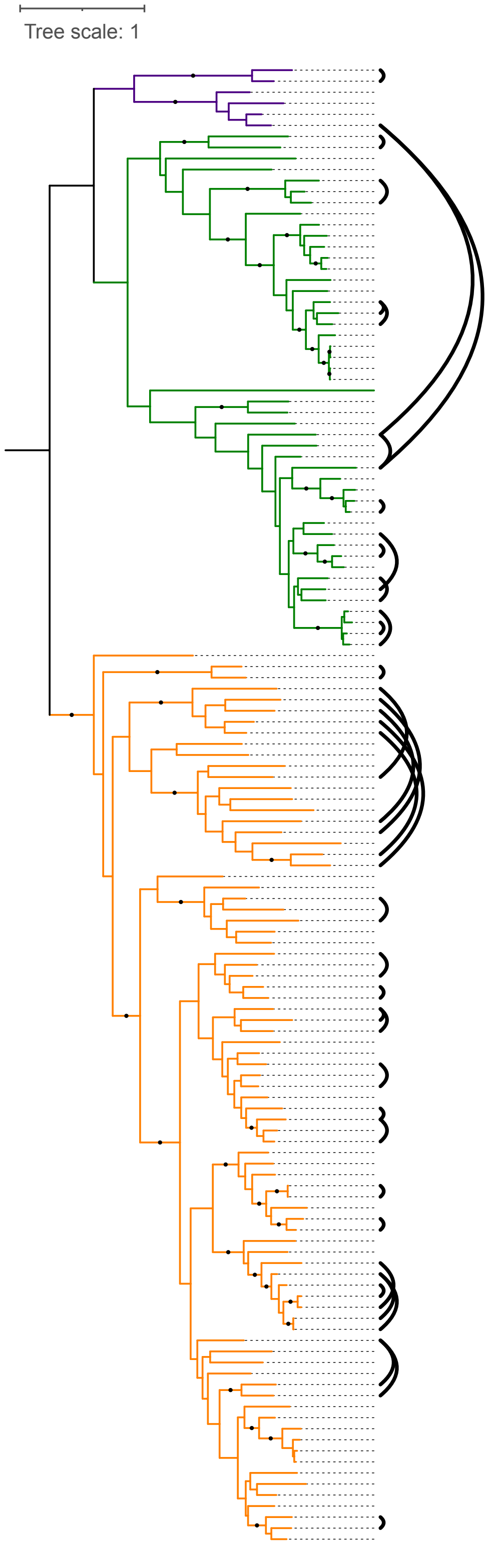

Supplemental Figure 3. Comparison of phylogenetic versus physical clustering of Col-0 NLRs. (Supports Figure 2)
Col-0 NLRome tree from Figure 2B with lines indicating genes within 5Kb.

Supplemental Data. Prigozhin and Krasileva (2021). Analysis of intraspecies diversity reveals a subset of highly variable plant immune receptors and predicts their binding sites. Plant Cell.

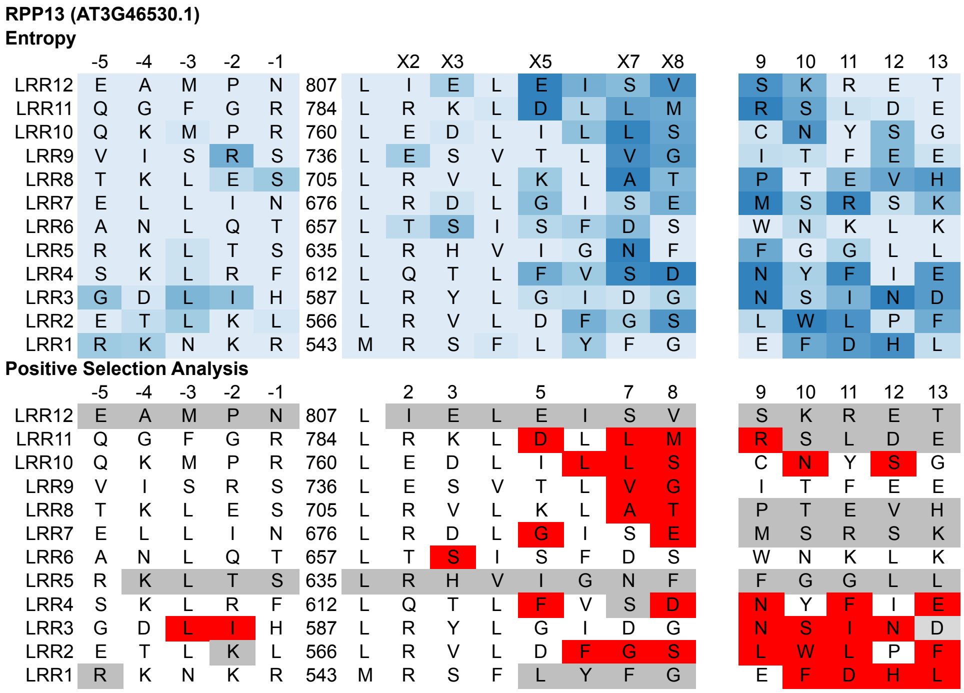

Supplemental Figure 4. Comparison of entropy-based and positive selection-based binding site predictions.
(Supports Figure 3)
2D surface representation of hvNLR RPP13 showing that high entropy residues are likely under positive selection. Top panel colored as in Figure 3A from low entropy (light blue) to high entropy (dark blue). Bottom panel highlights in red the residues under positive selection identified with paml (>95% posterior probability). Grey background residues were not analyzed due to presence of gaps in the alignment.
