# Supplementary material for: Analysis of intraspecies diversity reveals a subset of highly variable plant immune receptors and predicts their binding sites
Source: Plant Cell. 2021 Jan 25;33(4):998–1015. doi: 10.1093/plcell/koab013 (PMC8226289; doi:10.1093/plcell/koab013)
Supplement: koab013_Supplementary_Data [file tpc.00673.2020-s07.pdf › tpc.00673.2020-s07.pdf]

Analysis of intraspecies diversity reveals a subset of highly variable plant immune receptors and predicts their binding sites

Daniil M. Prigozhin and Ksenia V. Krasileva

Corresponding author: Ksenia V. Krasilevan kseniak@berkeley.edu

Review timeline:

**TPC2020-RA-00673**	Submission received:	Aug. 21, 2020
	1st Decision:	Oct. 2, 2020 *revision requested*
**TPC2020-RA-00673R1**	1st Revision received:	Dec. 18, 2020
	2nd Decision:	Dec. 28, 2020 *acceptance pending, sent to science editor*
	Final acceptance:	Jan. 8, 2021

REPORT: (The report shows the major requests for revision and author responses. Minor comments for revision and miscellaneous correspondence are not included. The original format may not be reflected in this compilation, but the reviewer comments and author responses are not edited, except to correct minor typographical or spelling errors that could be a source of ambiguity.)

TPC2020-RA-00673 1st Editorial decision – *revision requested* **Oct. 2, 2020**

As you will read in the review, the approach and results you present in the manuscript are viewed as quite interesting and likely to spur more experimental work on the structure and function of hvNLRs. However, there are concerns about the lack of experimental validation of the inferences drawn from your computational analyses. Reviewer 1 and to a lesser extent reviewer 2 would like to see some experimental validation added to the manuscript before publication. We would like for you to respond to the comments from all three reviewers, either adding experimental validation or strengthening your review of published work supporting your predictions. Would it be possible to include the unpublished data from the cited dissertation on RPP1-NdA function in this manuscript? Doing so would help TPC readers better appreciate the predictive power of molecular evolutionary analyses.

-- Reviewer comments:

Reviewer #1 (Comments for the Author):

The manuscript from Prigozhin and Krasileva focuses on the intraspecies diversity of plant NLR immune receptors and the prediction of their binding sites. They used Shannon entropy to calculate the sequence diversity of allelic NLRs and identified the residues that display high sequence polymorphisms. These polymorphic residues may account for direct effector recognition. Combining with the phylogenetic analysis, they defined some of the NLR subfamilies as "hvNLRs" that show high sequence variation. The prediction may also reflect whether the NLRs in each subfamily adopt direct or indirect mechanisms in detecting pathogen effectors. Overall, the concepts and computational pipeline developed are of interest to the community. However, the hypothesis proposed and conclusions lack experimental supports. Although the authors discussed other previous NLR studies that fit their observation, it is difficult to judge whether this is a robust method that makes informative predictions when a naïve NLR is used. Please see below for my detailed comments:

1. Combining phylogenetic information and Shannon entropy calculation is very interesting. It does show that some of the subfamilies display the "hv" feature whereas others do not. The authors claimed that these highly polymorphic residues predicted with this method are involved in effector binding. However, this claim lacks direct experimental evidence in this paper. For example, in the case of RPP1, in which the author has previously shown interaction with ATR1 through the LRR domain using co-IP experiments, can the authors test the idea proposed in this manuscript by mutating some of those residues? It would be interesting to know whether mutating these residues affect effector binding or cell death mediated by RPP1.

2. Similar case as in RPP13, since the author defined that RPP13 is a "hvNLR" that act through direct binding, can the author test the idea that whether RPP13 directly associates with ATR13 and whether mutations of these predicted residues impair the interaction?
3. The authors claim that RPP13 and ZAR1 emerged from the same hvNLR common ancestor. However, this claim is not convincing to me. First, is there an evolutionary study showing that ZAR1 and RPP13 are from a well-supported monophyletic group? That is, in fact, not supported in figure 2B. We probably accept that CNLs are from a common ancestor, but, from other studies with plant NLRome, the split between ZAR1 clade and other CNL clades appeared to be very ancient. To my knowledge, it is not well resolved whether the ZAR1 clade is closer to, for example, the RPM1 clade or RPP13 clade in the phylogenetic tree in figure 2B. Second, if they are from a common hvNLR ancestor, what happens to the other CNL clades such as RPP8, RPP7 as well as RPP39, and others? Are those polymorphic resides on the same surface as described in RPP13? Or are they from a different ancient hvNLRs?
4. Several of the sentences have issues of over claiming, please consider rephrasing these sentences:
Line 36: The author identified a limited number of NLR subfamilies that show high allelic diversity rather than showing by any experimental evidence that they are "responsible for generating new specificity".
Line 368: as mentioned above, this is still lacking direct evidence.
Line 435: given the points I raised above, the conclusion here is premature.
Line 529: The author did indicate that DM NLRs are within the hvNLR subfamily. However, given the fact that DM requires two incompatible genes and are allele-specific, the current method is far from making predictions on incompatible NLR loci.
5. Line 301: This claim is too general and is without sufficient experimental evidence provided in this manuscript.

Reviewer #3 (Comments for the Author):

The manuscript by Prigozhin and Krasileva utilises the power of pan-genome sequencing data to generate new, and support existing, hypotheses regarding NLR gene evolution. Importantly the work provide novel avenues towards future engineering strategies of NLRs, however, as acknowledged by the authors the success of this would be dependent on access to sufficient sequencing analysis of closely related specifies. Inevitably this will become more readily available in the near future and I therefore feel that the approach taken will be useful to the community at large. Overall, the work is very well written. The figures are informative (I particularly liked the 2D representation of LRR surfaces presented in Figure 3) and well presented and the flow through the results make it an enjoyable manuscript to read. I believe the outcomes of the work are important and would be of broad interest to plant scientist. I think the target journal is appropriate.

The results obtained are largely dependent on the phylogeny-based approach taken by the authors to separate NLRs into clades and cluster them further into near-allelic series to ultimately identify hvNLRs. As a non-expert in this area, I do not believe I am in a position to comment on (or review) the validity of the approaches taken to do this. Despite this, on face value it does appear that this approach was very successful, the separation into major clades (RPW8, CC and TIR) is achieved and the results presented in Figure 2, also appear to validate this approach. Figure 2 also help endorse the use of Shannon entropy plots and while the observations in Figure 2 are not particularly novel (much of the variation is in the LRR domain as to be expected), a lot less variation in helper NLR (for example), I feel this further validates overall the methods and approach taken. This is also well argued and described well in both the Introduction and Discussion. I'm generally in favour of the work and its publication but I do have some suggestions that I believe should be considered prior to publication.

Major consideration.
The biggest criticism I have of the work is that the authors do not provide any experimental validation based on the outcomes of the work presented in Figure 3/Supplemental Data Set 2. To some extent this is understandable, as many of the identified hvNLRs don't have known Avrs, but I think it would have really added to the paper to make that extra step. The authors do state in this section that the predictions are in agreement with previously published work for RPP13 and RPP1. The RPP1 work refers to one of the authors PhD thesis whereby they showed "D896K/S897Y/Y899R, allowed RPP1-NdA to recognize ATR1-Maks9, the effector allele that in wild type

combination is not recognized" but this work is not as easily accessible as a published paper. I would suggest that the authors consider articulating this in text. Preferably, they could identify the overlap of their results and the examples in a figure format (could include a model of the LRRs and those residues highlighted). This would also be useful for RPP13 (already a model available) and RPP1.

Reviewer #4 (Comments for the Author):

In the submitted manuscript, the authors carry out an analysis of Nucleotide-Binding Leucine Rich Repeat (NLR) receptors in Arabidopsis. They carry out a phylogenetic analysis of the family in Arabidopsis, assess sequence variability, identify highly variable NLRs (hvNLRs) and establish links between hvNLRs and known autoimmune loci. Using the experimentally determined structure of the NLR protein ZAR1, they used in silico methods to predict a structure for the RPP13 ligand-binding site. Using this, they examine the position of high entropy residues in RPP13. The authors also examine the position of hvNLRs within the phylogeny of Brachypodium NLRs. Overall, the analysis is comprehensive and interesting. I am not knowledgeable enough about plant immune receptors to comment on the novelty of the results but overall the phylogenetic analysis, entropy measures for sequence variability and the locating of the highly variable residues within the 3D structure are all well conceived and convincing.

Major Comments
1. I think the choice of phylogenetic approach is good, as is the decision to perform it manually. Whereas automated software (such as OrthoMCL) may be good at performing whole-genome orthogroup analysis, an expert curated analysis directed towards one specific gene family, especially one whose sequence evolution may be atypical, should ensure equal or higher accuracy.
2. I feel that additional species beyond Arabidopsis ecotypes in the NLR gene tree could have clarified a number of points:
3. I find the method for splitting the clades understandable, but somewhat arbitrary. By not including any other species in the gene tree, the authors have no benchmark for the evolutionary timescale over which various clades diverged and e.g. which diverged over a similar period in time. With additional species all clades diverging since the MRCA of the Brassicaceae, for example, could have been compared on a like-for-like basis (or some other suitable phylogenetic level). This would have made more precise claims amount rapidly evolving clades and highly variable NLRs. As it stands, the clades analysed could be of vastly different ages. Such limitations should be discussed.
4. The claim is made that, "this phylogeny... supporting previous observations of monophyletic origin of TNLs, CNLs and RNLs (Shao et al., 2016; Tamborski and Krasileva, 2020)". This relies on choosing the root for the tree used in the previous studies (between TNLs & non-TNLs), the authors have not independently verified this root and therefore that the root doesn't fall within one of the TNL, CNL and RNL groups. However, it should be noted that a different root would affect at most one of the 223 non-singleton clades analysed in the remainder of the paper.
5. Similarly, "These observations are consistent with NLR genes experiencing rapid shifts in the selection pressure". This is hard to judge without an understanding of the evolutionary period over which these changes took place.
6. Are there some genes missing from the phylogeny, for example AT1G33560 & AT5G66890 from the RNL clade?
7. hvNLRs are defined as those with 10 or more sites exceeding 1.5 bits of entropy and some general patterns are described. I think it would be helpful to show a histogram of this data for the clades analysed i.e. number of sites exceeding this cut-off. This would allow readers to assess the validity of the cut-off chosen.

TPC2020-RA-00673R1 1st Revision received Dec. 18, 2020

Reviewer comments and **author responses**:

Reviewer #1:

The manuscript from Prigozhin and Krasileva focuses on the intraspecies diversity of plant NLR immune receptors and the prediction of their binding sites. They used Shannon entropy to calculate the sequence diversity of allelic NLRs and identified the residues that display high sequence polymorphisms. These polymorphic residues may account for direct effector recognition. Combining with the phylogenetic analysis, they defined some of the NLR

subfamilies as "hvNLRs" that show high sequence variation. The prediction may also reflect whether the NLRs in each subfamily adopt direct or indirect mechanisms in detecting pathogen effectors. Overall, the concepts and computational pipeline developed are of interest to the community. However, the hypothesis proposed and conclusions lack experimental supports. Although the authors discussed other previous NLR studies that fit their observation, it is difficult to judge whether this is a robust method that makes informative predictions when a naïve NLR is used. Please see below for my detailed comments:

RESPONSE: Thank you for the thoughtful comments. We agree that our presented method constitutes predictions, and we are heartened by the strong overlap of highly variable residues in RPP1 with the contact residues in the recently published RPP1-ATR1 structure (Ma et al, 2020). Undoubtedly, more structures will provide better confidence intervals for the sequence-based predictions, and methods that will combine structural predictions with sequence diversity data will likely provide further improvement for both precision and recall.

Point 1. Combining phylogenetic information and Shannon entropy calculation is very interesting. It does show that some of the subfamilies display the "hv" feature whereas others do not. The authors claimed that these highly polymorphic residues predicted with this method are involved in effector binding. However, this claim lacks direct experimental evidence in this paper. For example, in the case of RPP1, in which the author has previously shown interaction with ATR1 through the LRR domain using co-IP experiments, can the authors test the idea proposed in this manuscript by mutating some of those residues? It would be interesting to know whether mutating these residues affect effector binding or cell death mediated by RPP1.

RESPONSE: The recent elucidation of RPP1-ATR1 structure allowed us to directly observe the overlap between contact residues and high-entropy amino acid positions, and to estimate a useful range (0.8 bits to 1.8 bits) of entropy cutoff values. In this range, lower cutoffs lead to better recall and the higher cutoffs result in better precision. We added new Figure 6 and a new paragraph to results and methods sections to discuss these observations.

Point 2. Similar case as in RPP13, since the author defined that RPP13 is a "hvNLR" that act through direct binding, can the author test the idea that whether RPP13 directly associates with ATR13 and whether mutations of these predicted residues impair the interaction?

RESPONSE: We have added experimental data on RPP13 to test our methods. Specifically, we have tested the contribution of 21 residues with entropy scores above 1.5 bits have shown that these residues are required for RPP13-Nd recognition of ATR13-Emco5 in a transient coexpression assay. Transfer of these residues alone to RPP13-Col however was not sufficient to achieve recognition while transfer of the whole region between aa 501 and 729 was. This experiment is therefore consistent with the RPP1 structure-based observation that high entropy cutoff values have high precision (we discover many true positives and few false positives) but will miss some contact residues. The tradeoff between precision and recall is expected and future experiments should show what cutoffs are practical for loss of function versus gain of function experiments.

Point 3. The authors claim that RPP13 and ZAR1 emerged from the same hvNLR common ancestor. However, this claim is not convincing to me. First, is there an evolutionary study showing that ZAR1 and RPP13 are from a well-supported monophyletic group? That is, in fact, not supported in figure 2B. We probably accept that CNLs are from a common ancestor, but, from other studies with plant NLRome, the split between ZAR1 clade and other CNL clades appeared to be very ancient. To my knowledge, it is not well resolved whether the ZAR1 clade is closer to, for example, the RPM1 clade or RPP13 clade in the phylogenetic tree in figure 2B. Second, if they are from a common hvNLR ancestor, what happens to the other CNL clades such as RPP8, RPP7 as well as RPP39, and others? Are those polymorphic resides on the same surface as described in RPP13? Or are they from a different ancient hvNLRs?

RESPONSE: Thank you for pointing this out. Indeed, the precise relationship of RPP13 and ZAR1 remains inconclusive. We agree that alternative interpretations are possible. For example, it is just as likely that a conserved common ancestor of ZAR1 and RPP13 had undergone a duplication, the products of which then only episodically experienced diversification and copy number variation, eventually arriving at the current state of things. We have edited this section accordingly.

We have included a reference in text to the recent study by the Kamoun lab focused on the evolutionary history of

ZAR1, which indeed appears to be an ancient NLR. Interestingly, some of the receptors in the ZAR1 clade have undergone recent diversification, suggesting that high variability can be an acquired rather than conserved feature of a receptor clade.

In Figure 2, RPP13 is the closest hvNLR to ZAR1, even though they diverged a very long time ago. RPP8 and RPP7 emerge from the same common ancestor, while RPP39 does not. To track the relationship between phylogeny and predicted binding sites, we added an additional Figure 4. It appears from that comparison that even across clades, related NLRs have similar binding sites. This suggests that information from the hvNLRs might help narrow down the candidate binding site residues of related low variability receptors. Clearly, more experimental work (both structures and mutagenesis) will be needed to test this inference.

Point 4. Line 368: as mentioned above, this is still lacking direct evidence.

RESPONSE: Last sentence in the hydrophobicity/PAML paragraph is changed to "Thus, results of the entropy analyses of hvNLR surfaces are consistent with the results of the widely accepted molecular evolution analyses performed on the underlying nucleotide sequences."

Point 5. Line 435: given the points I raised above, the conclusion here is premature.

RESPONSE: We agree that 'conclusion' is a strong word here, however we think that our interpretation is valid and can be included as a discussion point. We re-phrased this sentence accordingly. The sentence now reads "Our analyses suggest that in a given species diversity generation occurs in a limited subset of NLR genes creating a wide recognition potential, including binding to endogenous plant proteins."

Point 6. Line 529: The author did indicate that DM NLRs are within the hvNLR subfamily. However, given the fact that DM requires two incompatible genes and are allele-specific, the current method is far from making predictions on incompatible NLR loci.

RESPONSE: We removed the sentence on line 529, as we see it can be misinterpreted and it does not add to the final conclusions. We respectfully disagree though that hvNLRs cannot predict DMs. We observed that all known DMs fell into hvNLR subclades, and therefore hvNLRs can contain other previously unknown DMs. This information will be useful in mapping DMs in the future and prioritizing candidate genes for testing. It is true that hvNLRs represent only one of two components in incompatibility loci and are not going to predict their interacting partner. We also do not want to claim that we can predict exact alleles, similar to the exact functional alleles, cannot be predicted from hvNLRs.

Point 7. Line 301: This claim is too general and is without sufficient experimental evidence provided in this manuscript.

RESPONSE: We believe that this statement is now better supported by the analysis of the RPP1-ATR1 structure.

Reviewer #3:

Point 1. The biggest criticism I have of the work is that the authors do not provide any experimental validation based on the outcomes of the work presented in Figure 3/Supplemental Data Set 2. To some extent this is understandable, as many of the identified hvNLRs don't have known Avrs, but I think it would have really added to the paper to make that extra step. The authors do state in this section that the predictions are in agreement with previously published work for RPP13 and RPP1. The RPP1 work refers to one of the authors PhD thesis whereby they showed "D896K/S897Y/Y899R, allowed RPP1-NdA to recognize ATR1-Maks9, the effector allele that in wild type combination is not recognized" but this work is not as easily accessible as a published paper. I would suggest that the authors consider articulating this in text. Preferably, they could identifying the overlap of their results and the examples in a figure format (could include a model of the LRRs and those residues highlighted). This would also be useful for RPP13 (already a model available) and RPP1.

RESPONSE: We have undertaken the suggested validation of predictions via i) evaluation of the recently determined RPP1/ATR1 structure adding new Figure 6 and its accompanying paragraphs and ii) targeted mutagenesis experiments on RPP13 expanding Figure 5 and the accompanying text to incorporate the results and discussion of these findings. In both cases we observed that high entropy residues are required for binding and/or recognition.

Reviewer #4:

Point 1. I think the choice of phylogenetic approach is good, as is the decision to perform it manually. Whereas automated software (such as OrthoMCL) may be good at performing whole-genome orthogroup analysis, an expert curated analysis directed towards one specific gene family, especially one whose sequence evolution may be atypical, should ensure equal or higher accuracy.

RESPONSE: Thank you for this comment. We directly compared OrthoMCL results with our approach, and found that crucial information was lost due to "fine slicing" of rapidly diverging sequences in several NLR clades.

Point 2. I feel that additional species beyond Arabidopsis ecotypes in the NLR gene tree could have clarified a number of points:

RESPONSE: Thank you for this important suggestion. We have added NLR sequences from *Capsella rubella* and *Arabidopsis lyrata* using the Evolutionary Placement Algorithm to the NLRome tree in Supplemental Figure 1 and tracked presence of *C. rubella* and *A. lyrata* sequences in the initial NLR clades, summarized in Supplemental Data Set 1. This addition allowed us to preserve the well-resolved clades in the original *A. thaliana* phylogeny while providing the necessary point of comparison. We find that the best way to view this data is to use iTOL prune feature to zoom in to individual nodes within the panNLRome tree. We have added the following text "To gain insight into the relative age of the initial clades we used the Evolutionary Placement Algorithm to place Arabidopsis lyrata and Capsella rubella NLRs in the A. thaliana pan-NLRome (Supplemental Figure 1). Of the initial 65 clades, 53 had representative sequences from either or both outgroups (Supplemental Data Set 1)."

Point 3. I find the method for splitting the clades understandable, but somewhat arbitrary. By not including any other species in the gene tree, the authors have no benchmark for the evolutionary timescale over which various clades diverged and e.g. which diverged over a similar period in time. With additional species all clades diverging since the MRCA of the Brassicaceae, for example, could have been compared on a like-for-like basis (or some other suitable phylogenetic level). This would have made more precise claims amount rapidly evolving clades and highly variable NLRs. As it stands, the clades analysed could be of vastly different ages. Such limitations should be discussed.

RESPONSE: We found the placements did not provide a good basis for splitting the initial clades due to the fact that the added species are likely undergoing the same gene gain/loss/diversification processes as *A. thaliana*. For example, initial clade Int10969_308 containing *RPP5*, *RPP4*, *SNC1*, and five other genes in ecotype Columbia had one *A. lyrata* and 3 *C. rubella* sequences mapped to it. It was refined to 11 subclades with as few as 2 and as many as 101 sequences per clade. Another interesting example is initial clade Int9836_17, which had no Columbia representation, was absent from *A. lyrata*, but got a *C. rubella* hit.

It appears that to accurately date various subclades, many more outgroup species with multiple ecotypes need to be added to guard against frequent gene loss. The pan-NLRome comparison across closely related species might therefore be very interesting, but is outside the scope of the present work. As a result, we have tried to guard against using "rapid/recent diversification" and the like as we agree with the reviewer that such statements need stronger support.

Point 4. The claim is made that, "this phylogeny... supporting previous observations of monophyletic origin of TNLs, CNLs and RNLs (Shao et al., 2016; Tamborski and Krasileva, 2020)". This relies on choosing the root for the tree used in the previous studies (between TNLs & non-TNLs), the authors have not independently verified this root and therefore that the root doesn't fall within one of the TNL, CNL and RNL groups. However, it should be noted that a different root would affect at most one of the 223 non-singleton clades analysed in the remainder of the paper.

RESPONSE: We agree and have edited the text and figure legend to indicate that we placed the root arbitrarily on the TNL/non-TNL branch to simplify downstream analysis. The relevant segment now reads "... this phylogeny clearly split into clades corresponding to the three canonical architectures: RPW8, Coiled-coil, and TIR domain-containing NLRs (Shao et al., 2016; Tamborski and Krasileva, 2020). We arbitrarily placed the root of the tree between TNL and non-TNL NLRs to simplify downstream analyses."

Point 5. Similarly, "These observations are consistent with NLR genes experiencing rapid shifts in the selection pressure". This is hard to judge without an understanding of the evolutionary period over which these changes took place.

RESPONSE: We agree with the reviewer and have edited the text accordingly. It now reads "These observations are consistent with closely related NLR genes experiencing different selection pressures."

Point 6. Are there some genes missing from the phylogeny, for example AT1G33560 & AT5G66890 from the RNL clade?

RESPONSE: We used a stringent 70% NB-ARC HMM coverage cutoff to simplify the analysis and excluded both the sequences with short NB-ARCs and short NB-ARCs from sequences with multiple NB-ARCs.

Point 7. hvNLRs are defined as those with 10 or more sites exceeding 1.5 bits of entropy and some general patterns are described. I think it would be helpful to show a histogram of this data for the clades analysed i.e. number of sites exceeding this cut-off. This would allow readers to assess the validity of the cut-off chosen.

RESPONSE: Thank you for the excellent suggestion. We have included the histogram of the number of sites over 1.5 bits per final clade alignment as Supplemental Figure 2.

TPC2020-RA-00673R1 2nd Editorial decision – *acceptance pending* Dec. 28, 2020

Thank you for the experimental and computational work included in your revised submission. We are pleased to inform you that your paper entitled "Intraspecies diversity reveals a subset of highly variable plant immune receptors and predicts their binding sites" has been accepted for publication in The Plant Cell, pending a final minor editorial review by journal staff. At this stage, your manuscript will be evaluated by a Science Editor with respect to its presentation of scientific content, compliance with journal policies, and presentation for a broad readership.

Final acceptance from Science Editor Jan. 8, 2021
